# Enhancing prestin oligomerization for superior sonogenetics

**DOI:** 10.7150/thno.122468

**Published:** 2026-01-01

**Authors:** Hsien-Chu Wang, Thi-Nhan Phan, Tzu-Han Chen, Yun Lin, Ning Hsu, Ching-Hsiang Fan, Po-Han Chiang, Chun-Hao Chen, Chih-Kuang Yeh, Yu-Chun Lin

**Affiliations:** 1 Institute of Molecular Medicine, National Tsing Hua University, Hsinchu 300044, Taiwan.; 2 Department of Biomedical Engineering and Environmental Sciences, National Tsing Hua University, Hsinchu 300044, Taiwan.; 3 Department of Biomedical Engineering, National Cheng Kung University, Tainan 701401, Taiwan.; 4 Medical Device Innovation Center, National Cheng Kung University, Tainan 701401, Taiwan.; 5 Center of Transformative Bioelectronic Medicine, College of Medicine, National Cheng Kung University, Tainan 701401, Taiwan.; 6 Institute of Intelligent Bioelectrical Engineering, National Yang Ming Chiao Tung University, Hsinchu 300093, Taiwan.; 7 Department of Electronics and Electrical Engineering, National Yang Ming Chiao Tung University, Hsinchu 300093, Taiwan.; 8 Institute of Molecular and Cellular Biology, National Taiwan University, Taipei 106319, Taiwan.; 9 Department of Biomedical Sciences and Engineering, Tzu Chi University, Hualien 970374, Taiwan.; 10 Department of Biomedical Engineering, Chung Yuan Christian University, Taoyuan 320314, Taiwan.; 11 Department of Medical Science, National Tsing Hua University, Hsinchu 300044, Taiwan.

**Keywords:** sonogenetics, ultrasound, prestin, neuromodulation, *Caenorhabditis elegans* behavior

## Abstract

**Background:** Sonogenetics enables non-invasive control of cellular activity using ultrasound (US)-responsive proteins. Prestin, a motor protein from outer hair cells, can render cells responsive to US when ectopically expressed, offering potential therapeutic applications for neurodegenerative diseases and epilepsy. However, current prestin variants exhibit suboptimal oligomerization, reducing their efficacy in neuromodulation. This study aimed to enhance prestin sonogenetic performance by improving oligomerization.

**Methods:** We screened four echolocating bat prestin orthologs and point mutants of mouse prestin variants for enhanced US responsiveness. Subcellular distribution and oligomerization of prestin variants were evaluated using live-cell imaging and Förster resonance energy transfer. Calcium imaging was used to assess functional responses in HEK293T cells and cultured neurons. *In vivo* functionality was tested in mice by AAV-mediated expression of prestin variants in hippocampal neurons and motor cortex, followed by transcranial US stimulation and c-Fos staining to assess neuronal activation. To evaluate cross-species efficacy, we expressed prestin variants in ray sensory neurons of *Caenorhabditis elegans* and quantified US-evoked behavioral responses. Tissue safety was confirmed via histological analysis in mice.

**Results:** Echolocating bat prestins did not improve US responsiveness compared to mouse prestin. In contrast, an engineered variant, Prestin(N548S, V715G), exhibited enhanced oligomerization and punctate membrane localization. This variant triggered stronger Ca²⁺ influx *in vitro*. *In vivo*, US stimulation of the cortex expressing Prestin(N548S, V715G) induced robust neuronal activation, as indicated by increased c-Fos expression and evoked tail movement in mice. In *Caenorhabditis elegans*, expression of Prestin(N548S, V715G) in ray neurons conferred significant US-evoked behavioral responses. No adverse histological effects were observed in mouse tissue.

**Conclusion:** Our findings show that enhancing prestin oligomerization, rather than relying on naturally evolved echolocating variants, significantly improves its sonogenetic efficacy. The Prestin(N548S, V715G) variant represents a potent and biosafe tool for non-invasive neuromodulation and holds promise for future therapeutic applications in neurological disorders.

## Introduction

Medical ultrasound (US), sound waves with frequencies above 20 kHz, has recently been used as an external stimulus to manipulate cellular activities 1-3. It fulfills the need for non-invasive manipulation of targeted cells buried in deep tissues due to its remarkable penetrability, safety, and spatiotemporal resolution 3. Several US sensors, either naturally existing or exogenously introduced into targeted cells, are responsive to medical US approaches collectively called sonogenetics 1-5. These powerful tools have successfully enabled non-invasive neuromodulation to improve symptoms in Parkinson's disease 4,6, epilepsy 7,8, tumors 9, and others 1,5,10. Although still in experimental stages, they hold great potential for future clinical translation.

Genetically encoded sensors play vital roles in sonogenetics, as they confer sensitivity to distinct bioeffects triggered by medical US 5. Among these sensors, Piezo1, Piezo2 11,12, TRPV4 13,14, MEC-4 15,16, TRPA1 17,18, TRPC1, TRPP2, and TRPM4 19 are abundant in distinct tissues, resulting in differences in sensitivity and responses to US stimulation across tissues. To trigger desired effects in targeted cells, the introduction of mutated sensors with improved sensitivity or proteins from evolutionarily divergent species, as well as optimized US parameters for orthogonal sensors, is required. For example, GvpA/C (Gas vesicle protein A/C), derived from cyanobacteria and haloarchaea, can form gas vesicles that respond to acoustic pressure in mammalian cells 20-22. MscL-I92L and MscL-G22S, two *E. coli* mechanosensitive channel variants of large conductance carrying mutations known to enhance mechanosensitivity, respond to US stimulation at defined parameters 4,22-25. With these orthogonal sensors, US can efficiently stimulate target cells genetically modified with minimal background effects in naive cells.

Prestin, a voltage-sensitive motor protein in cochlear outer hair cells, plays an important role in high-frequency hearing in mammals 26. By comparing Prestin sequences across echolocating and non-echolocating species, researchers found two significant amino acid substitutions: N7T and N308S. These evolutionary amino acid substitutions are prevalent in echolocating species but are often absent in non-echolocating species. Introducing N7T and N308S mutations into the mouse prestin gene significantly increased its sensitivity to US 5,27,28. Mechanistically, this enhanced sensitivity is linked to the formation of Prestin clusters—an oligomeric state closely associated with its electromotility, cochlear amplification, and US sensitivity 29-31. Prestin(N7T, N308S)-mediated sonogenetics serves as a powerful approach to ameliorate the symptoms of Parkinson's disease and suppress epileptic seizures in rats 6,8. We here aimed to explore and engineer prestin variants for better US sensitivity.

## Methods

### Cell Culture

HEK293T cells (ATCC CRL-11268) and HeLa cells (ATCC CCL-2) were maintained at 37 °C in a humidified atmosphere containing 5% CO_2._ HEK293T cells were cultured in Dulbecco's Modified Eagle's Medium (DMEM; Corning, 10-013-CV) supplemented with 10% (v/v) fetal bovine serum (FBS; Corning, 35-010-CV), 1% penicillin, and 1% streptomycin (Corning, 30-002-CI). Primary cortical neurons were isolated from embryonic day 18 (E18) Sprague-Dawley rat embryos obtained from the National Center for Biomodels (NCB, NIAR, Taiwan). Dissected cortices were washed in Hank's Balanced Salt Solution (HBSS) without calcium and magnesium (Gibco, 14185-052) supplemented with 10 mM HEPES (Gibco, 15630-080) to minimize contamination. Tissues were then enzymatically dissociated in a digestion solution containing 0.6 mg/mL papain (Sigma-Aldrich, 76220), 0.6 mg/mL DNase I (Sigma-Aldrich, DN25), 0.2 mg/mL L-cysteine (ThermoFisher Scientific, 173600250), 1.5 mM CaCl₂ (Biomatik, A2306), and 0.5 mM EDTA (Invitrogen, AM9260G) in HBSS at 37 °C for 15-20 min with gentle agitation. The enzymatic reaction was quenched with 10% horse serum (Gibco, 16050) in Neurobasal medium (Gibco, A35829-01). Neurons were dissociated by gentle trituration (20-30 times) using a 10 mL plastic pipette (Fisherbrand, 13-678-11E), filtered through a 40 µm cell strainer (Corning, 352340) to remove debris, and collected by centrifugation at 1000 × g for 5 min at room temperature. The resulting pellet was resuspended in Neurobasal medium supplemented with 1× GlutaMAX™ (Gibco, 35050-061), 12.5 μM L-glutamic acid (Sigma-Aldrich, G1251), 1% penicillin-streptomycin, and 2% B-27™ supplement (Gibco, A35828-01). Neurons were seeded at a density of 8 × 10⁵ cells per well on poly-L-lysine-coated 6-well plates and maintained at 37 °C with 5% CO₂. One-third of the culture medium (excluding L-glutamic acid) was replaced with fresh medium every 3-4 days.

### Plasmid Construction

The pEGFP-C1 vector (Takara Bio) was used as the backbone for constructing plasmids encoding R-GECO and Prestin. R-GECO, a red fluorescent genetically encoded calcium indicator, was cloned for live-cell imaging applications. The full-length coding sequence of mouse Prestin (MM-Prestin; GenBank accession number NM_001289787) was subcloned into a Venus expression vector using the *XhoI* and *BamHI* restriction sites, resulting in a Venus-tagged MM-Prestin construct. For the Förster Resonance Energy Transfer (FRET) experiment, the CFP-tagged MM-prestin construct was generated by replacing Venus with CFP using the *NheI* and *BglII* restriction enzymes. To generate functional variants, specific point mutations—N7T, N308S, N548S, and V715G—were introduced into MM-Prestin using site-directed mutagenesis. For cross-species comparative analysis, prestin genes from four echolocating bat species—*Aselliscus stoliczkanus* (AS), *Megaderma spasma* (MS), *Miniopterus fuliginosus* (MF), and *Rhinolophus ferrumequinum* (RF)—were codon-optimized and synthesized (IDT). To introduce sequence diversity, the Diversify® PCR Random Mutagenesis Kit (Takara, #630703) was employed with the following thermal cycling conditions: initial denaturation at 94 °C for 30 s; 30 cycles of 94 °C for 30 s, 57 °C for 30 s, and 68 °C for 3 min; followed by a final extension at 68 °C for 3 min. The mutagenized prestin PCR products were then subcloned into mammalian expression vectors containing a CMV promoter using the NEBuilder® HiFi DNA Assembly Master Mix (New England Biolabs, M5520AA) to enable efficient expression in transfected cells.

### DNA Transfection

HEK293T cells were transfected with plasmid DNA using the LT-1 transfection reagent (Mirus Bio, MR-MIR2300), following the manufacturer's protocol. Briefly, plasmid DNA and LT-1 were mixed at a ratio of 1 µg DNA to 3 µL LT-1 in 40 µL of Opti-MEM (Gibco, 31985-070) and incubated at room temperature for 20 min to allow DNA-lipid complex formation. Meanwhile, HEK293T cells were harvested by trypsinization, washed once with phosphate-buffered saline (PBS; Corning, 21-040-CV), and pelleted by centrifugation at 2000 rpm for 3 min. The cell pellet was then resuspended in fresh DMEM containing 10% FBS. The transfection mixture was gently added to the cell suspension and mixed thoroughly. Cells were seeded onto poly-L-lysine (0.2 mg/mL, Sigma-Aldrich, P2636)-coated 25-mm glass coverslips placed in six-well plates (GeneDireX, PC306-0050) or onto Lab-Tek 8-well chamber slides (ThermoFisher Scientific, 155411), and incubated at 37 °C in a humidified incubator with 5% CO₂ for 1-2 h. After cell attachment, each well was supplemented with additional DMEM containing 10% FBS. Transfected cells were incubated for 21-24 h prior to imaging or subcellular distribution analysis.

### Gene delivery in Primary Cultured Cortical Neurons

Recombinant adeno-associated virus serotype 9 (AAV9) encoding Venus-MM-Prestin(N548S, V715G) or Venus alone was produced by the AAV core facility at Academia Sinica, Taiwan. On day *in vitro* 4 (DIV4), primary cortical neurons were transduced with AAVs encoding either CB-Venus or CB-Venus-MM-Prestin(N548S, V715G). Following 24 h of incubation at 37 °C, the virus-containing medium was replaced with a 1:1 mixture of conditioned and fresh culture medium. For long-term maintenance, half of the culture medium was routinely replaced with Neurobasal medium supplemented with B-27. Fourteen days post-infection, fluorescence imaging was performed using a Nikon T*i*-E inverted microscope.

For transfection of four bat prestins into primary neurons, including Venus-AS-Prestin, Venus-MS-Prestin, Venus-MF-Prestin and Venus-RF-Prestin, the teleofection (CaP-based calcium phosphate transfection) mixture was prepared by following the previously published protocol 32 with mixing 6 μL of 2 M CaCl_2_, 2 μg of DNA, and appropriate H_2_O with a final volume of 200 μL (6-well plate). Then the CaP nanoparticle-cargo mixture was obtained by adding 200 μL of 2 × HeBS dropwise to a cargo and CaCl_2_ mixture with a continuously gentle vortex (Vortex Mixer/GENIE2, power 4). Then 800 μL of Neurobasal medium was taken from the cultured cells and added into the CaP nanoparticle-cargo mixture with approximately 10 pipetting cycles without vortexing to get a teleofection medium. Retain the remaining culture medium in the wells of 6-well plates for further incubation after teleofection. The transfection mixture was dropped into culture neurons gently, and neurons were then incubated at 37 °C, 5% CO_2_ incubator for 60-70 min. After appropriate incubation, remove the teleofection medium and wash with warm HBSS three times. The original culture medium was returned into cultured neurons for subsequent incubation. Fourteen days post-infection, fluorescence imaging was performed using a Nikon T*i*-E inverted microscope.

### Live-Cell Imaging

Calcium imaging was conducted using a Nikon T*i*-E inverted fluorescence microscope equipped with a 20 × air objective lens (Nikon) and a DS-Qi2 CMOS camera (Nikon). Cells were maintained at 37 °C in a 5% CO₂ atmosphere within a temperature- and humidity-controlled chamber (Live Cell Instrument) to preserve physiological conditions throughout the imaging process. To examine the subcellular localization of Venus or Venus-MM-Prestin constructs in HEK293T cells or HeLa cells, imaging was performed using a Nikon T*i*-E paired with a 60 × oil immersion objective lens (Nikon). Z-stack images were acquired with a step size of 0.3 µm across 17 optical sections (FRET images were acquired across 11 optical sections). Maximum z-projections were generated using the built-in deconvolution module of the NIS-Elements AR software (Nikon). Quantitative analysis of prestin puncta number and size was carried out using ImageJ. For ultrafast imaging of prestin puncta dynamics, a Nikon T*i*-E equipped with a high-sensitivity camera (Prime 95B, Photometrics) and a 60 × objective was used to capture high temporal resolution images. This setup enables real-time monitoring of puncta vibration and potential mechanosensitive responses in transfected cells.

### Animal Experiments

All animal experiments were conducted using male C57BL/6JNarl (B6) mice aged 5-8 weeks and weighing 16-25 g, obtained from the National Center for Biomodels (NCB, NIAR, Taiwan). The mice were housed in a controlled environment with a 12-h light/dark cycle (lights on at 07:00), at a temperature of 22-23 °C and relative humidity of 30-70%. Food and water were available ad libitum. All procedures involving animals were reviewed and approved by the Institutional Animal Care and Use Committee (IACUC) of National Tsing Hua University and were carried out in accordance with the NIH Guide for the Care and Use of Laboratory Animals (IACUC approval number: NTHU110072).

### *C. elegans* Strains

All worms used in this study carried the *him-5(e1409)* mutation, which increases the number of males in the population 33. To generate transgenic *C. elegans* lines, the CMV-Venus-MM-Prestin(N548S, V715G) construct was used as a template to amplify an *AgeI*-Venus-MM-Prestin(N548S, V715G)-*XhoI* fragment by PCR. This fragment was then subcloned into the *AgeI*/*XhoI*-digested Ppkd2-GFP plasmid. The pkd2 promoter drives expression specifically in ray neurons, which are located in the fan-like structure of the adult male tail and are involved in modulating mating behavior 34. Transgenic lines were established using standard microinjection techniques 35 and designated as: [*Pmyo-2-GFP*, *Ppkd2-Venus-MM-Prestin(N548S, V715G)* + *him-5(e1409)*]. Extrachromosomal arrays were injected at the following concentrations: Pmyo-2-GFP (20 ng/μL, used as a co-injection marker) and Ppkd2-Venus-MM-Prestin(N548S, V715G) (80 ng/μL).

### FUS Setup and Parameter Examination

For *in vitro* experiments, FUS was delivered using a single-element transducer (V318-SU, Olympus Panametrics; center frequency: 0.5 MHz), driven by a function generator (AFG3022C, Tektronix, Beaverton, OR, USA) and a radio-frequency power amplifier (Model 240L, Electronics & Innovation, Ltd.). Pulsed US was applied with the following parameters: 0.5 MPa acoustic pressure, 2000 cycles per burst, 10 Hz PRF (pulse repetition frequency), and a total stimulation duration of 3 s. To ensure efficient acoustic coupling, a detachable water cone filled with degassed water was attached to the transducer, with its tip submerged in the culture medium. During real-time calcium imaging, the US beam was aligned confocally with the microscope objective to maintain precise stimulation within the imaging field 27.

For *in vivo* FUS stimulation, a custom-designed single-element geometrically focused transducer was used, incorporating a piezoceramic disc (diameter: 19.9 mm; radius of curvature: 20 mm; focal depth: 20 mm). US pulses were delivered at a center frequency of 0.5 MHz and a peak negative pressure of 0.5 MPa, with 2000 cycles per burst, and a 10 Hz PRF. Each stimulation lasted 5 min, corresponding to a mechanical index (MI) of 0.71. The signal was generated by a function generator and amplified using a radio-frequency amplifier (Model 2100L, Electronics & Innovation, Rochester, NY, USA). The acoustic field's spatial profile was characterized in a degassed water tank using a needle-type hydrophone (HNC-0400, ONDA, Sunnyvale, CA, USA), revealing a focal zone of 4 mm in diameter and 22 mm in axial length 8. Transcranial targeting of the hippocampus or motor cortex was achieved using a computer-controlled three-axis translation system. The selected FUS parameters were based on prior optimization studies for effective activation of neurons expressing Venus-MM-Prestin(N7T, N308S) 27,28.

For worm stimulation, *C. elegans* microinjected with Ppkd2-Venus-MM-Prestin(N548S, V715G) were placed on NGM agar pads featuring a 2-mm central hole filled with 10 μL of M9 buffer (Figure 5A) 36. FUS was applied using the same custom-built transducer described above, with a total stimulation duration of 2 min. US pulses were generated by a function generator (AFG3022C, Tektronix, Beaverton, OR, USA) and amplified through a radio-frequency power amplifier (Model 150A100C, Amplifier Research, Souderton, PA, USA). FUS stimulation was delivered between 30 and 150 s during a 5-min video recording using a camera (DFK 33UX250, The Imaging Source, NC, USA). The agarose pad containing the worm was positioned directly above the transducer, allowing US to propagate through the agar medium to the worm (Figure 5A). The FUS parameters were selected based on previous studies that identified optimal conditions for activating Venus-MM-Prestin-expressing cells 6,8,27,28. Prior to stimulation, young adult male worms were transferred to NGM agar plates seeded with OP50 and cultured overnight in the absence of hermaphrodites.

### Cell Viability Test

To determine whether FUS stimulation induces cytotoxicity, cell viability was evaluated using the Cell Counting Kit-8 (CCK-8; Dojindo Laboratories, CK04-01), following the manufacturer's instructions. HEK293T cells expressing either Venus alone or Venus-MM-Prestin(N548S, V715G) were seeded at a density of 2.5 × 10⁵ cells per well on poly-L-lysine-coated 25-mm glass coverslips in six-well culture plates. After overnight incubation at 37 °C in a humidified atmosphere with 5% CO₂, cells were subjected to FUS stimulation using the following parameters: 0.5 MPa acoustic pressure, 2000 cycles per burst, 10 Hz PRF, and a total stimulation duration of 3 s.

Three hours after stimulation, 10 μL of CCK-8 reagent was added directly to each well, and the plates were incubated at 37 °C to allow the formation of the formazan product. Absorbance was measured at 450 nm using a microplate reader (800TS, BioTeK). All measurements were performed in triplicate. Cell viability was calculated as a percentage relative to the untreated control group.

### *In Vivo* Gene Delivery and FUS Stimulation

AAVs were stereotactically injected into the right hippocampus or motor cortex of 5-week-old male C57BL/6JNarl mice (n = 18 and 12 in hippocampus and motor cortex experiments, respectively). Mice were anesthetized with 3% isoflurane for induction and maintained at 1% throughout the procedure. Animals were positioned in a mouse stereotaxic frame (David Kopf Instruments), and the scalp was sterilized with povidone-iodine followed by 75% ethanol. Using a Hamilton microsyringe, 1.5 µL of either AAV-CB-Venus-MM-Prestin(N7T, N308S)(titer: 3.8 x 10^13^ vg/mL), AAV-CB-Venus-MM-Prestin(N548S, V715G)(titer: 9.4 × 10¹² vg/mL) or AAV-CB-Venus (titer: 5.9 × 10¹³ vg/mL) was injected into the hippocampus or motor cortex, targeting coordinates based on the Mouse Brain in Stereotaxic Coordinates (Paxinos and Franklin, 4^th^ edition), hippocampus region: AP = -2.0 mm, ML = -2.0 mm, DV = -2.0 mm and motor cortex region: AP = 0.5 mm, ML = -1.5 mm, DV = -1.5 mm. Injections were performed at a flow rate below 0.3 µL/min. To minimize viral backflow, the syringe was left in place for an additional 10 min before slow retraction. The incision was then closed with surgical sutures, and mice were placed under a warming lamp for recovery.

Three weeks after AAV injection, mice were randomly assigned to six groups (n = 3 per group): (1) Venus-MM-Prestin(N7T, N308S) with FUS stimulation, (2) Venus-MM-Prestin(N7T, N308S) without FUS, (3) Venus-MM-Prestin(N548S, V715G) with FUS stimulation, (4) Venus-MM-Prestin(N548S, V715G) without FUS, (5) Venus alone with FUS, and (6) Venus alone without FUS. FUS stimulation was delivered transcranially to the right hippocampus or motor cortex while the fur on the mouse head was removed to prevent any potential blocking of the stimulation delivery and secured in the stereotaxic apparatus for accurate targeting. The US parameters were as follows: 0.5 MHz center frequency, 0.5 MPa peak negative pressure, 2000 cycles per burst, 10 Hz PRF, and a total stimulation duration of 5 min.

### Mouse Tail Movement Recording

To conduct transcranial US stimulation of the intact motor cortex, mice were anesthetized with 1.5% isoflurane for induction and maintained at 0.5-1% throughout the procedure. The fur on the head was trimmed, the skin was incised, and the mouse was subsequently positioned on a custom-designed plastic platform in a prone posture with the tail freely hanging (Figure 5F), ensuring that the tail did not contact the platform to avoid interference with tail movement. The FUS transducer was lowered to positions above the skull corresponding to targeted brain regions using standard stereotactic coordinates, and US gel was applied between the transducer and skull. A camera was employed to record tail movement for 16 min, including before (1 min baseline), during (5 min FUS stimulation), and after (10 min post-stimulation) US stimulation. The movement displacement from high-resolution videos was analyzed using NIS-Elements software (Nikon, Tokyo, Japan) and further processed using GraphPad Prism 10 (GraphPad Software, San Diego, CA, USA).

### Immunohistochemistry Staining

Mice were euthanized either 2 h after FUS stimulation or three weeks post-AAV injection. Animals were deeply anesthetized with 3% isoflurane and perfused transcardially with 0.9% normal saline, followed by immersion in 4% paraformaldehyde overnight. Brains were extracted and cryoprotected sequentially in 10%, 20%, and 30% sucrose (each for 24 h), embedded in a water-soluble glycol-resin medium (Leica Biosystems, Surgipath, 38011480), and stored at -20 °C. Coronal brain sections (20 μm thick) were obtained using a cryostat.

For general histological evaluation, hematoxylin and eosin (H&E) staining (Leica Biosystems, Surgipath, 3801522 and 3801602) was used to assess tissue morphology and hemorrhage. Apoptosis was detected using the TUNEL assay (BrdU-Red, Abcam, ab66110). Microglial activation was assessed by immunostaining with a rabbit anti-Iba1 polyclonal antibody (1:100, ThermoFisher Scientific, PA5-21274) incubated overnight at 4 °C, followed by an Alexa Fluor® 594-conjugated goat anti-rabbit IgG secondary antibody (1:100, Abcam, ab150080) for 1 h at room temperature. Nuclei were counterstained with DAPI (Calbiochem, 268298). Iba1-positive cells and Iba1^+^/DAPI^+^ cell ratios were quantified using fluorescence microscopy.

To verify FUS-induced neuronal activation, c-Fos immunostaining was performed on brain sections collected 2 h after stimulation. Sections were permeabilized with 0.3% Triton X-100 (Sigma-Aldrich, T8787) for 15 min and blocked in PBS containing 5% goat serum and 5% bovine serum albumin (BSA) for 2 h at room temperature. Samples were incubated overnight at 4 °C with a rabbit anti-c-Fos antibody (1:750, Cell Signaling Technology, #2250) and a chicken anti-GFP antibody (1:500, GeneTex, GTX13970). Following PBS washes containing 0.3% Triton X-100, secondary antibodies were applied: Alexa Fluor® 594-conjugated goat anti-rabbit IgG (1:1000, Invitrogen, A11012) and Alexa Fluor® 488-conjugated goat anti-chicken IgG (1:1000, ThermoFisher Scientific, A11039), for 2 h at room temperature. Slices were mounted using Fluoroshield™ with DAPI mounting medium (GeneTex, GTX30920) and stored at -20 °C in the dark. Venus-MM-Prestin expression was confirmed by direct Venus fluorescence in the hippocampus. Neuronal activation was quantified by counting Venus⁺/c-Fos⁺ double-positive cells within defined regions of interest (ROIs; 450 μm × 450 μm). Two to three ROIs per section were analyzed and averaged. Quantifications were performed using samples from at least three animals. All image acquisition and analysis were performed in a blinded manner.

### Worm Tail-Curling Assay

Based on a previously described method 37, we quantified tail posture to evaluate the curling response during mating behavior. Tail curvature was analyzed using the NIS-Elements AR software. Movements that resembled curling were first annotated, and the software was then used to identify the frame showing maximal curvature. In this frame, the radius of curvature was obtained by fitting the tail to an arc of a circle. To account for the extent of tail involvement, the curvature value was adjusted by multiplying the radius by the ratio *c/tc*, where c denotes the circumference of the fitted circle (π × diameter) and tc is the length of the tail segment contacting the circle. Movements with an adjusted curvature value < 20 were classified as curling events.

### Worm Survival Rate Assay

Following FUS stimulation, worms were transferred from the agar pad to NGM agar plates seeded with OP50 and cultured for 2 days. Survival was then assessed by counting the number of live worms. The survival rate was calculated using the following formula:

Survival rate (%) = (Number of surviving worms / Total number of initial worms) × 100

### Imaging Data Analysis and Statistical Analysis

Fluorescence image data, including real-time calcium signal intensity, were analyzed using the NIS-Elements software (Nikon, Tokyo, Japan). Regions of interest (ROIs) were manually selected, and time-series signal intensities were extracted and normalized to enable comparison across experimental conditions. FRET data were also analyzed using NIS-Elements, with CFP-MM-Prestin(N548S, V715G) designated as the donor and Venus-MM-Prestin(N548S, V715G) as the acceptor. The resulting FRET signals were visualized as pseudo-colored rainbow images. Statistical analyses were performed using GraphPad Prism 10 (GraphPad Software, San Diego, CA, USA). An F-test was first conducted to assess the equality of variances between groups. Depending on the result, unpaired two-tailed Student's *t*-tests were applied to evaluate statistical significance. For comparisons involving multiple groups, significance was determined relative to the appropriate control. All data are presented as mean ± standard error of the mean (S.E.M); mean ± S.D. is used where specified. A *P*-value of less than 0.05 was considered statistically significant. All imaging experiments were conducted in a blinded manner. Cell cultures and imaging fields were randomly assigned to experimental groups, and data acquisition was performed under identical imaging conditions. Each experiment was independently repeated at least three times. Detailed sample sizes and statistical outcomes are provided in the corresponding figure legends.

## Results

### Characterization of US Sensitivity in Sonar Bat Prestins

Our previous studies introduced two amino acid substitutions—N7T and N308S—frequently found in echolocating but not in non-echolocating species, into the mouse prestin gene. These mutations enhanced the US sensitivity of mouse prestin in HEK293T cells and the mouse brains 27,28, suggesting that prestins from echolocating species may inherently possess high US sensitivity. To test this hypothesis, we selected four echolocating bat species whose Prestin sequences have been widely used in comparative auditory research and are representative of distinct evolutionary lineages associated with high-frequency hearing 26,38. We cloned prestin genes from four echolocating bat species 26,38: *Aselliscus stoliczkanus* (AS), *Megaderma spasma* (MS), *Miniopterus fuliginosus* (MF), and *Rhinolophus ferrumequinum* (RF). These genes (hereafter referred to as AS-Prestin, MS-Prestin, MF-Prestin, and RF-Prestin) all encode threonine at position 7, while serine at position 308 is present only in MS- and MF-Prestins (Figure S1A). To assess their distribution and clustering properties, we fused each prestin variant to Venus, a yellow fluorescent protein. Fluorescence imaging revealed that Venus-tagged AS-, MS-, and MF-Prestins form clusters in HEK293T cells, whereas RF-Prestin accumulated predominantly in the nucleus (Figure S1B). Consistent with previous reports 6,28, N7T and N308S mutants in *Mus musculus* Prestin (MM-Prestin) significantly enhanced clustering (Figure S1B and S1C). However, none of the four bat-derived Prestin variants formed more clusters than wild-type or mutant MM-Prestin in HEK293T cells (Figure S1B and S1C), suggesting that bat Prestins do not exhibit enhanced clustering in HEK293T cells.

To evaluate the US responsiveness of the bat Prestins, we co-expressed each candidate with a calcium biosensor, CFP (a cyan fluorescent protein)-R-GECO, in HEK293T cells. A single US pulse (0.5 MHz, 0.5 MPa, 10 Hz PRF, 2000 cycles per burst, 3-s duration) was applied to the cells. In line with our prior findings 6,28, only MM-Prestin(N7T, N308S), but not wild-type MM-Prestin, triggered calcium influx in response to US, as indicated by R-GECO fluorescence (Figure S2). Notably, cells expressing any of the four bat Prestins exhibited little or no US-induced calcium influx (Figure S2). We also transfected these four bat prestins into rat primary cortical neurons and demonstrated that they did not, or only weakly, trigger calcium influx in response to US (Figure S3). These results suggest that prestins from the four sonar bats tested do not confer US sensitivity to HEK293T cells or primary neurons. Given that Prestin clustering is critical for US responsiveness 28-31, we assume that this lack of clustering may underlie the inability of these bat Prestins to respond to US stimulation.

### Screening Prestin Variants with Enhanced Protein Clustering

**Figure 1:**
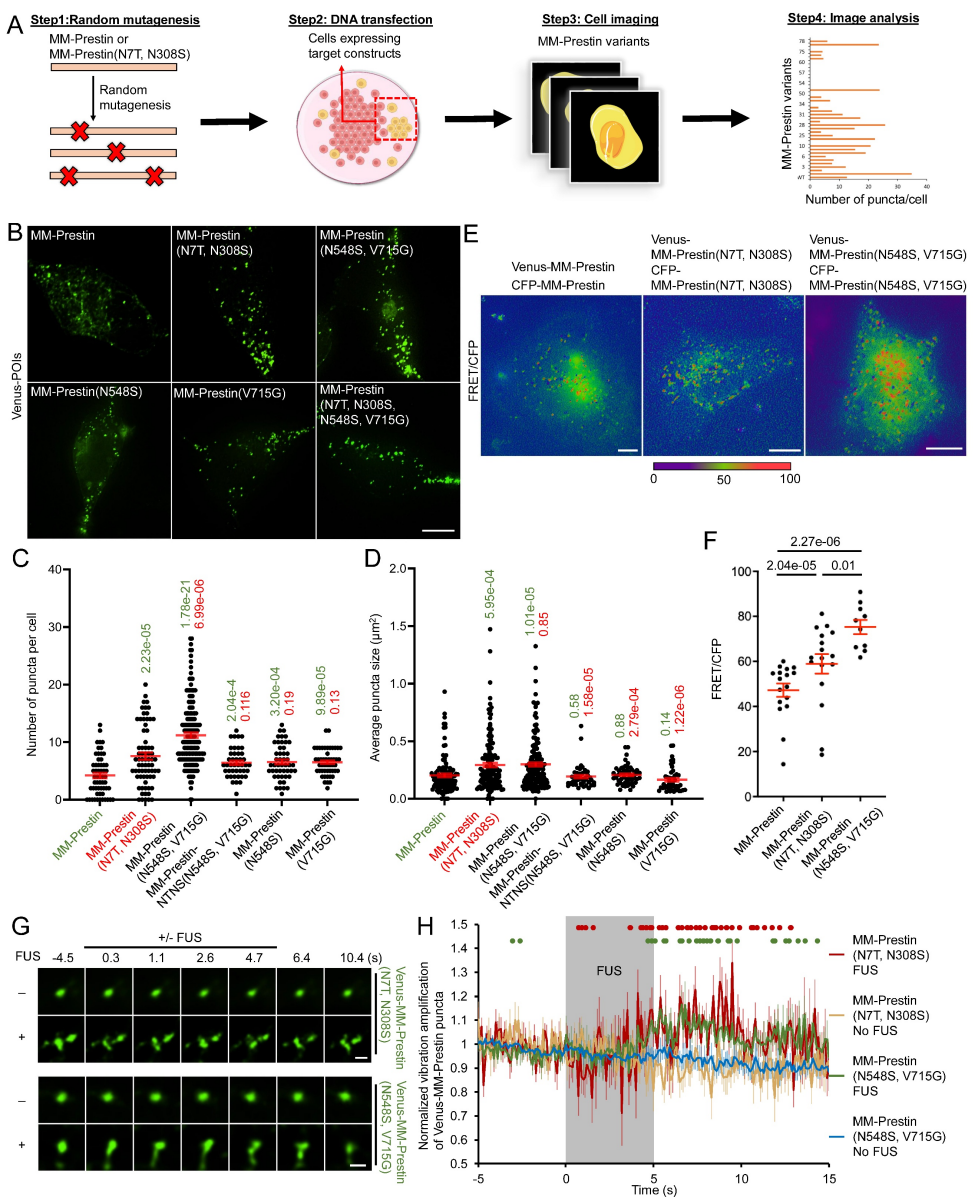
Screening of Prestin variants with enhanced puncta formation. (A) Schematic overview of the screening workflow for MM-Prestin variants exhibiting enhanced puncta formation. Random mutants of Venus-MM-Prestin or Venus-MM-Prestin(N7T, N308S) were generated using PCR-based mutagenesis. HEK293T cells were transfected with each mutant construct, followed by fluorescence imaging and quantitative analysis of puncta formation. (B) Representative fluorescence images of HEK293T cells expressing Venus-MM-Prestin, Venus-MM-Prestin(N7T, N308S), Venus-MM-Prestin(N548S, V715G), Venus-MM-Prestin(N548S), Venus-MM-Prestin(V715G) or Venus-MM-Prestin(N7T, N308S, N548S, V715G), respectively. Maximum z-projections were generated from 17 optical sections (0.3 µm step size). Scale bar: 10 µm. (C, D) Quantification of puncta number (C) and puncta size (D) in cells expressing the indicated constructs. Data (red) represent mean ± S.E.M. from three independent experiments; n = 57, 70, 159, 48, 49, and 43 cells (left to right) in (C); n = 111, 139, 153, 54, 47, and 43 cells (left to right) in (D). Student's *t*-test was used to compare each construct with MM-Prestin (green) or MM-Prestin(N7T, N308S) (red). (E) FRET imaging of HeLa cells co-transfected with CFP and Venus-tagged MM-Prestin, MM-Prestin(N7T, N308S), or MM-Prestin(N548S, V715G). Scale bars: 10 µm. (F) Quantification of FRET/CFP ratio in cells shown in (E). Data (red) represent mean ± S.E.M. from three independent experiments; n = 17, 17, and 10 cells (left to right). Student's *t*-test was used for statistical comparison. (G) Time-lapse images showing the structural dynamics of Venus-MM-Prestin(N7T, N308S) and Venus-MM-Prestin(N548S, V715G) puncta in cells with or without 0.5 MHz FUS stimulation. Scale bar: 1 µm. (H) Average vibration amplification of Venus-MM-Prestin(N7T, N308S) and Venus-MM-Prestin(N548S, V715G) with or without FUS stimulation (0.5 MHz, 0.5 MPa, 10 Hz PRF, 2000 cycles per burst, 5-s duration). Data represent mean ± S.E.M. from more than three independent experiments. n = 9 (MM-Prestin(N548S, V715G); FUS), 14 (MM-Prestin(N548S, V715G); no FUS), 8 (MM-Prestin(N7T, N308S); FUS), and 14 cells (MM-Prestin(N7T, N308S); no FUS). Student's *t*-test was used to determine significance between the +FUS and -FUS groups for each Prestin variant. Green (MM-Prestin(N7T, N308S)) and Red (MM-Prestin(N548S, V715G)) dots indicate time points showing significant differences (*p*<0.05) between FUS and no FUS conditions.

We next aimed to identify Prestin variants with improved US sensing. Although previous studies have reported that lipid composition 39, protein-protein interactions 40, and post-translational modifications 41 contribute to Prestin clustering and oligomerization, the detailed mechanisms underlying its clustering remain poorly understood. To address this, we generated random mutations in MM-Prestin or MM-Prestin(N7T, N308S) using PCR-directed mutagenesis and sought to identify variants with enhanced clustering capability. A total of 192 mutated MM-Prestin variants were transfected into HEK293T cells and screened via live-cell imaging (Figure 1A; Figure S4). Among them, MM-Prestin(N548S, V715G) exhibited the most pronounced consistently robust clustering capability (Figure 1B, C). Consistent with a previous study 28, quantification revealed that the N7T and N308S mutations significantly promoted punctate formation (Figure 1B, C). Notably, MM-Prestin(N548S, V715G) produced a higher number of puncta than both wild-type MM-Prestin and MM-Prestin(N7T, N308S) (Figure 1B, C), whereas the puncta size did not show further enlargement relative to MM-Prestin(N7T, N308S) (Figure 1B, D). Furthermore, both N548S and V715G mutations were required for this enhancement, as neither single mutant increased puncta formation compared to MM-Prestin(N7T, N308S) (Figure 1B-D). Förster Resonance Energy Transfer (FRET) imaging revealed that Venus- and CFP-tagged MM-Prestin(N548S, V715G) form dimers or oligomers in punctate regions (Figure 1E), and notably, this variant exhibited a stronger FRET signal than MM-Prestin(N7T, N308S), indicating more robust clustering of MM-Prestin(N548S, V715G) (Figure 1E, F). Interestingly, the combination of N548S/V715G with N7T/N308S did not further enhance puncta formation, and this quadruple mutant exhibited fewer and smaller puncta compared to MM-Prestin(N548S, V715G) and MM-Prestin(N7T, N308S) (Figure 1B, D). These findings suggest that the N7T/N308S and N548S/V715G mutations promote puncta formation through distinct mechanisms and do not act synergistically. Instead, combining the two sets of mutations appears to disrupt puncta assembly.

**Figure 2:**
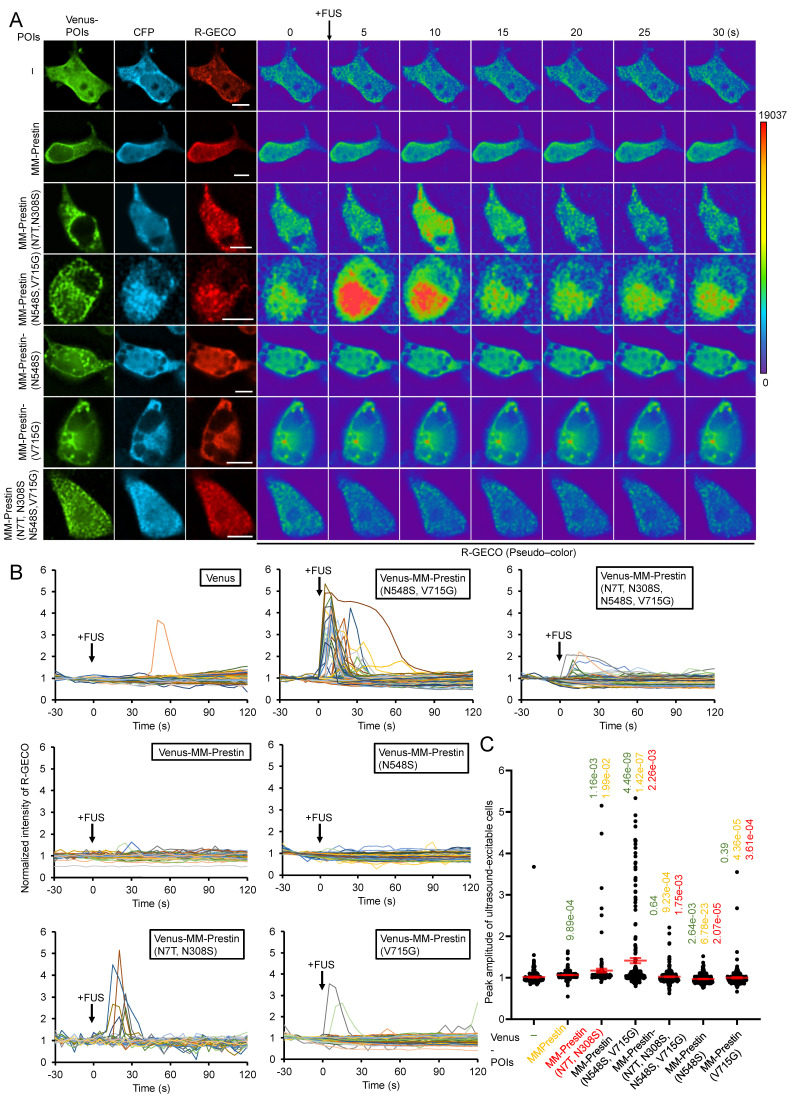
N548S and V715G double mutations enhance the US sensitivity of MM-Prestin. (A) HEK293T cells were co-transfected with CFP-R-GECO and the indicated constructs. Twenty-four hours post-transfection, a pulse of FUS (0.5 MHz, 0.5 MPa, 10 Hz PRF, 2000 cycles per burst, 3-s duration) was applied. R-GECO fluorescence intensity was monitored in real time (pseudo-color). Scale bar: 10 µm. (B) Time course of normalized R-GECO fluorescence intensity in cells expressing the indicated constructs following 0.5 MHz FUS stimulation. Data were collected from more than three independent experiments: n = 233, 242, 136, 213, 249, 225, and 234 cells for Venus, Venus-MM-Prestin, Venus-MM-Prestin(N7T, N308S), Venus-MM-Prestin(N548S, V715G), Venus-MM-Prestin(N7T, N308S, N548S, V715G), Venus-MM-Prestin(N548S), and Venus-MM-Prestin(V715G), respectively. (C) Peak R-GECO fluorescence amplitude upon FUS stimulation in cells expressing the indicated constructs. Data (red) represent mean ± S.E.M. from more than three independent experiments. Student's *t*-test was used for statistical comparison with Venus alone (green), MM-Prestin (orange) or MM-Prestin(N7T, N308S) (red), respectively.

We then evaluated the US sensitivity of MM-Prestin(N548S, V715G). Short-pulse US robustly triggered calcium influx in HEK293T cells transfected with MM-Prestin(N548S, V715G) (Figure 2A, B). The peak amplitude of calcium signals in responsive cells indicated that MM-Prestin(N548S, V715G) induced significantly stronger calcium influx compared to Venus alone, MM-Prestin, and MM-Prestin(N7T, N308S) (Figure 2C). Importantly, single mutations at either N548S or V715G were insufficient to elicit a comparable response (Figure 2C). Ultrafast imaging further revealed that MM-Prestin(N7T, N308S) and MM-Prestin(N548S, V715G)-positive puncta vibrated upon US stimulation. (Figure 1G, H)-a phenomenon associated with calcium response 28. However, the average vibration amplification per punctum was comparable between the two variants (Figure 1H). The greater abundance of puncta in MM-Prestin(N548S, V715G) likely contributes to its overall improved US sensitivity.

We also tested whether FUS affects cell viability using the CCK-8 assay. The FUS parameters used throughout these experiments did not affect the viability of cells expressing either Venus alone or Venus-MM-Prestin(N548S, V715G) (Figure S5). Together, these results demonstrate that the N548S/V715G double mutation enhances both the clustering and US responsiveness of MM-Prestin.

### MM-Prestin(N548S, V715G) Potentiates US Sensitivity in Primary Neurons

**Figure 3:**
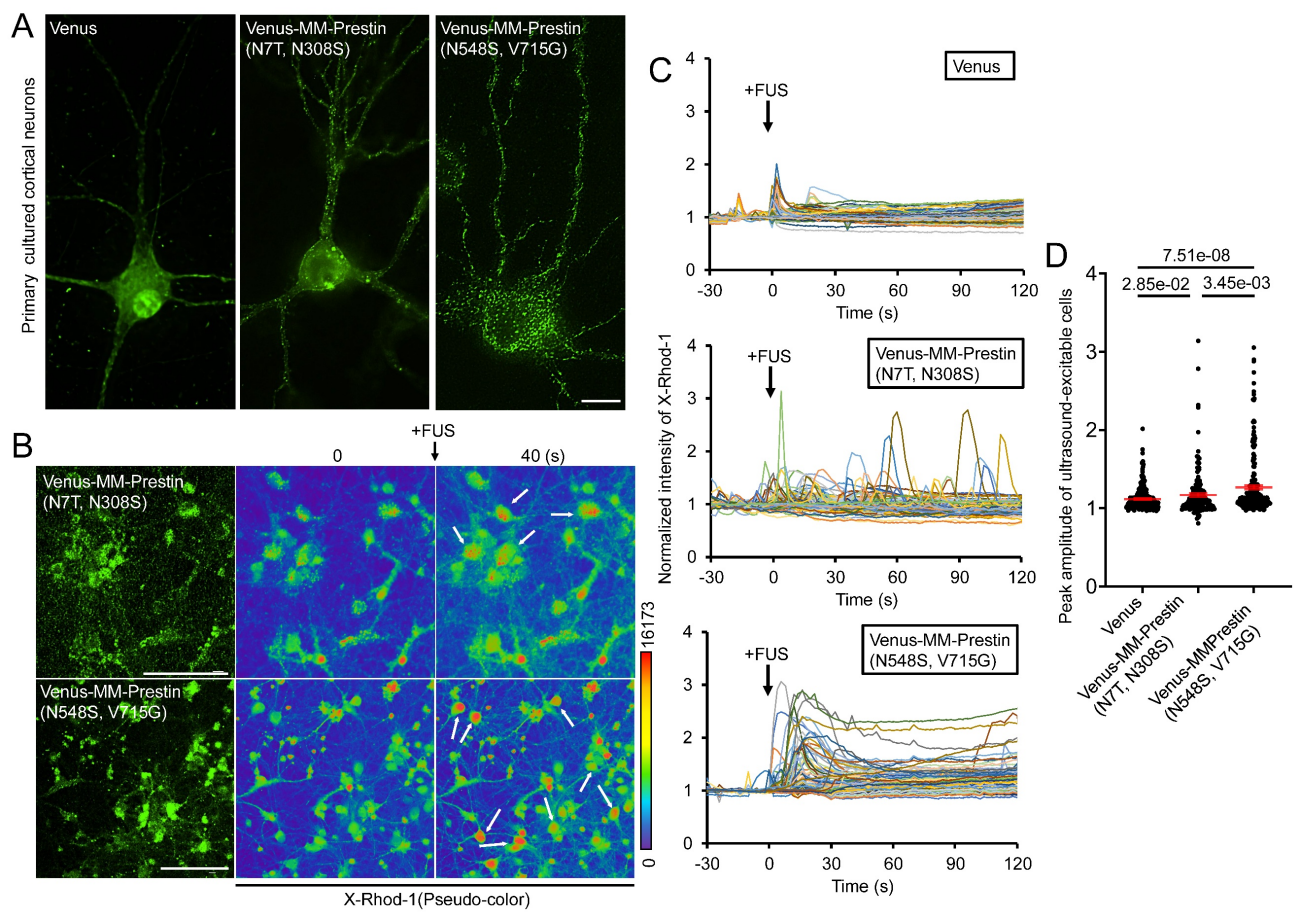
MM-Prestin(N548S, V715G) enhances US sensitivity in primary neurons. (A) Rat primary cortical neurons were infected with AAV encoding either Venus, Venus-MM-Prestin(N7T, N308S), or Venus-MM-Prestin(N548S, V715G). Protein expression and localization were assessed after 48-h infection period. Maximum intensity z-projections were generated from 11 stacks, each spaced 0.3 µm apart. Scale bar: 10 µm. (B) Neurons infected with AAV-Venus-MM-Prestin(N7T, N308S) or AAV-Venus-MM-Prestin(N548S, V715G) were stained with the calcium indicator X-Rhod-1. Upon pulsed FUS stimulation (0.5 MHz, 0.5 MPa, 10 Hz PRF, 2000 cycles per burst, 3-s duration), X-Rhod-1 fluorescence intensity (pseudo-color) was monitored using live-cell imaging. Activated neurons are indicated by arrows. Scale bar: 100 µm. (C) Time course of X-Rhod-1 fluorescence intensity in neurons expressing the indicated constructs following 0.5 MHz FUS stimulation. Data were collected from more than three independent experiments: n = 255, 180, and 227 cells for the Venus, Venus-MM-Prestin(N7T, N308S), and Venus-MM-Prestin(N548S, V715G) groups, respectively. (D) Peak X-Rhod-1 fluorescence intensity from C. Data (red) represent mean ± S.E.M. from more than three independent experiments. Student's *t*-test was used for statistical comparison between indicated groups.

We next examined whether MM-Prestin(N548S, V715G) confers US sensitivity to neurons. Embryonic rat primary cortical neurons were infected with adeno-associated viral (AAV) vectors encoding either Venus alone, Venus-MM-Prestin(N7T, N308S), or Venus-MM-Prestin(N548S, V715G). Immunofluorescence imaging showed that Venus-MM-Prestin(N548S, V715G) formed puncta efficiently in primary neurons, indicating that its clustering ability is not limited to HEK293T cells (Figure 3A). Moreover, Venus-MM-Prestin(N548S, V715G) formed puncta more robustly and abundantly than MM-Prestin(N7T, N308S), further demonstrating its superior clustering capacity (Figure 3A). Calcium responses were assessed using X-Rhod-1 staining. While cortical neurons expressing Venus-MM-Prestin(N7T, N308S) or Venus-MM-Prestin(N548S, V715G) were imaged using the red-fluorescent calcium indicator, X-Rhod-1. A short US pulse induced only moderate calcium influx in Venus-expressing neurons (Figure 3C, D). Expression of Venus-MM-Prestin(N7T, N308S) significantly increased both the magnitude and probability of calcium influx (Figure 3C, D). Notably, Venus-MM-Prestin(N548S, V715G) elicited an even stronger and more reliable calcium response upon FUS stimulation, surpassing the effects of Venus-MM-Prestin(N7T, N308S) (Figure 3C, D). These findings confirm that MM-Prestin(N548S, V715G) acts as a superior US actuator that forms puncta in primary neurons and enhances their responsiveness to pulsed US stimulation.

### MM-Prestin(N548S, V715G) Confers US Sensitivity in Mouse Brains

**Figure 4:**
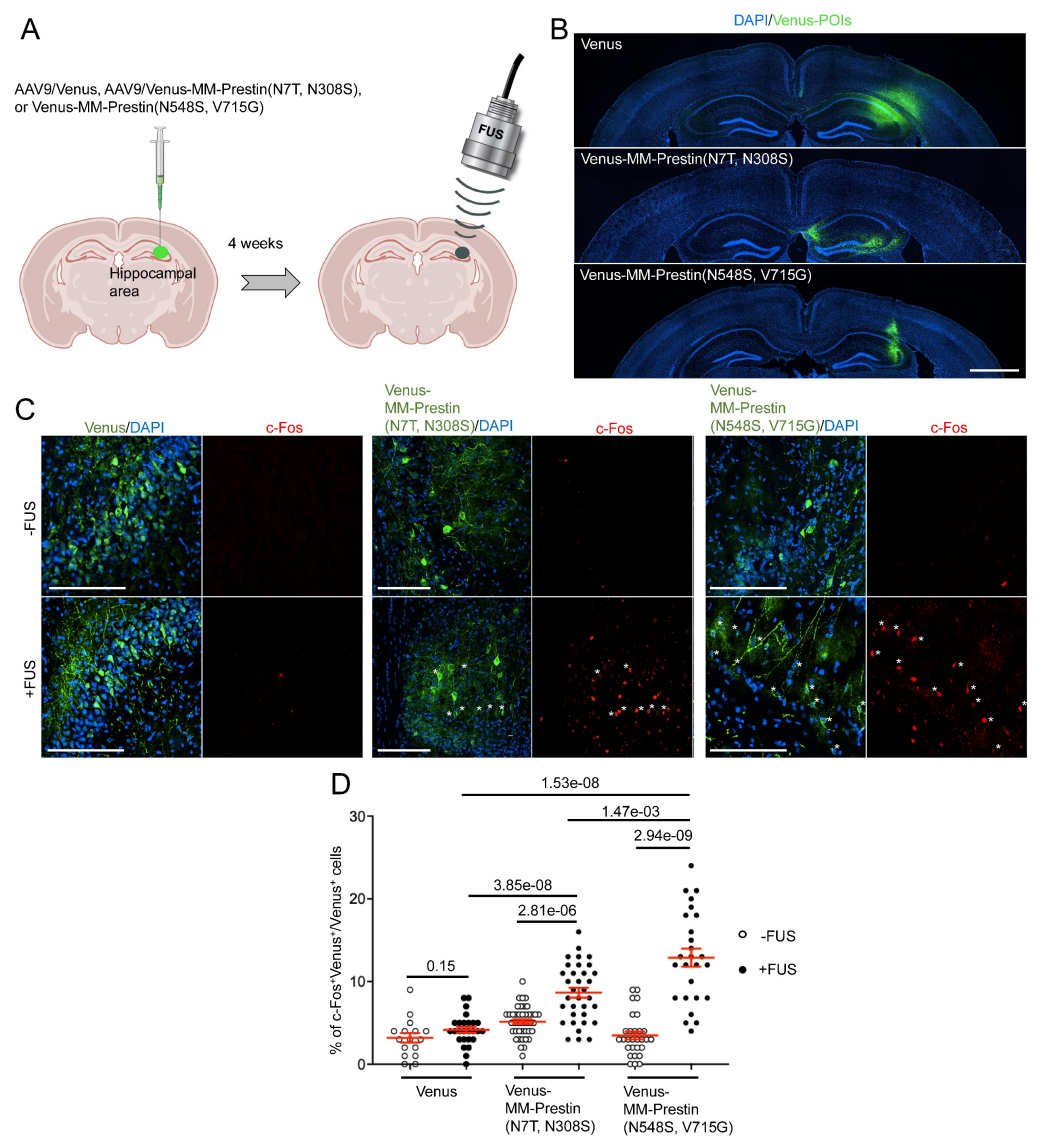
Transcranial FUS evokes stronger activation of hippocampal neurons expressing MM-Prestin(N548S, V715G) compared to MM-Prestin(N7T, N308S). (A) Schematic illustration of the in vivo transcranial FUS stimulation targeting the hippocampus. AAV9 encoding Venus, Venus-MM-Prestin(N7T, N308S), or Venus-MM-Prestin(N548S, V715G) was stereotactically injected into the hippocampal region of mouse brains. Three weeks after AAV injection, transcranial FUS was non-invasively applied to hippocampal area. (B) Representative brain sections showing unilateral injection of AAV9-Venus, AAV9-Venus-MM-Prestin(N7T, N308S) or AAV9-Venus-MM-Prestin(N548S, V715G) into the hippocampus. Scale bar: 1 mm. (C) Representative images of hippocampal brain sections with or without FUS stimulation. Neuronal activity was assessed via immunostaining of c-Fos (red) in neurons expressing Venus, Venus-MM-Prestin(N7T, N308S), or Venus-MM-Prestin(N548S, V715G) following FUS stimulation (0.5 MHz, 0.5 MPa, 10 Hz PRF, 2000 cycles per burst, 5-min duration). Asterisks indicate c-Fos⁺/Venus⁺ double-positive neurons. Scale bar: 100 μm. (D) Quantification of the percentage of c-Fos-positive neurons among Venus-expressing cells, with or without FUS stimulation. Data (red) represent mean ± S.E.M. from 16-42 sections, derived from more than 3 mice per condition. Student's *t*-test was used to assess statistical significance.

To evaluate whether MM-Prestin(N548S, V715G) enables non-invasive neuronal activation *in vivo*, we tested its function in the mouse brain. AAV9 vectors encoding either Venus, Venus-MM-Prestin(N7T, N308S), or Venus-MM-Prestin(N548S, V715G) were stereotactically injected into the right hippocampal region of mouse brains (Figure 4A). Expression at the injection sites was confirmed by immunohistochemistry (Figure 4B). To assess functional activation, we examined whether US stimulation could induce expression of c-Fos, an immediate early gene and marker of neuronal activity, in neurons expressing Venus-MM-Prestin(N7T, N308S) or Venus-MM-Prestin(N548S, V715G). Quantitative analysis showed that US stimulation did not increase the number of c-Fos-positive neurons in mice injected with AAV9-Venus (Figure 4C, D). Moreover, expression of Venus-MM-Prestin(N7T, N308S) or Venus-MM-Prestin(N548S, V715G) alone did not elevate c-Fos levels compared to Venus controls, indicating minimal spontaneous activation in the absence of US (Figure 4C, D). Consistent with previous studies 6,28, FUS stimulation robustly induced c-Fos expression in neurons expressing Venus-MM-Prestin(N7T, N308S), confirming its ability to mediate FUS-responsive neuronal activation *in vivo*. Notably, Venus-MM-Prestin(N548S, V715G) elicited an even stronger c-Fos response, demonstrating that this engineered prestin variant enables more effective neuromodulation *in vivo* (Figure 4C, D).

### Sonogenetic Modulation of Animal Behavior Using MM-Prestin(N548S, V715G)

**Figure 5:**
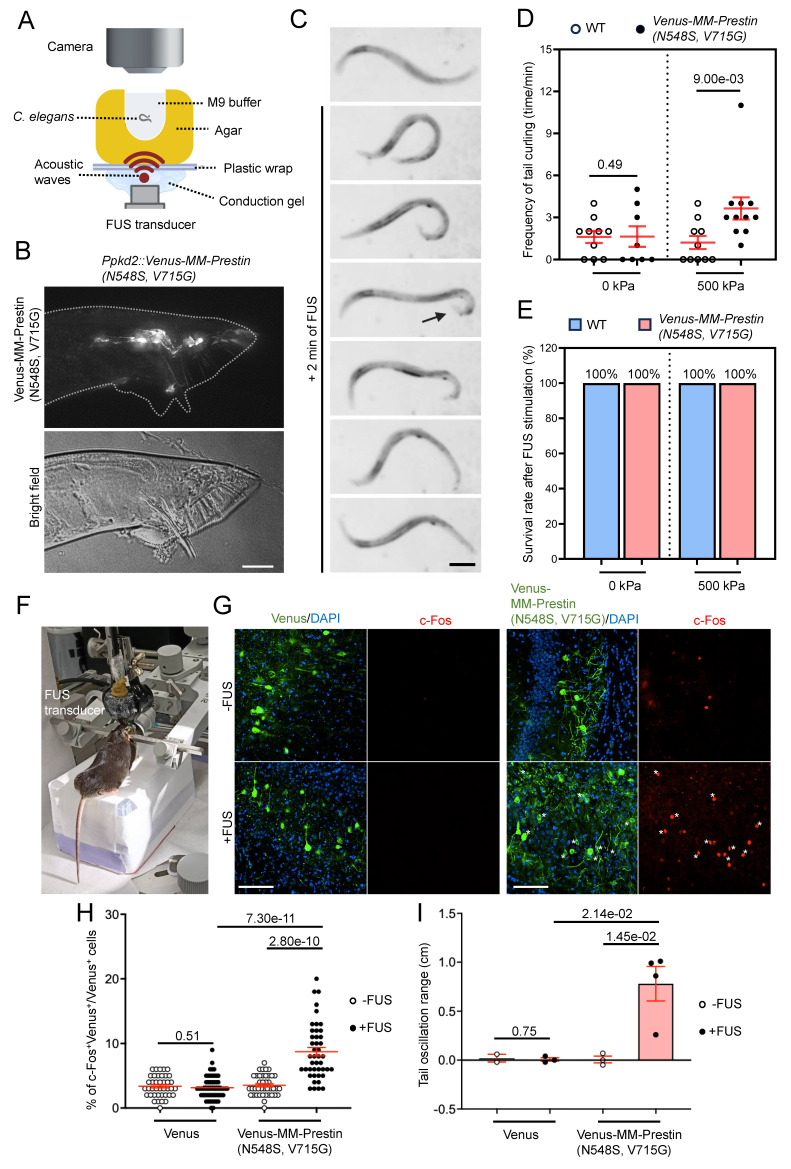
FUS controls the behavior of different species through MM-Prestin(N548S, V715G). (A) Schematic of the FUS-stimulation setup. The agar containing the worm is placed on plastic wrap, which is fixed on stage. The FUS transducer is placed under agarose gel and delivers FUS (0.5 MHz, 0.5 MPa, 10 Hz PRF, 2000 cycles per burst, 2-min duration) through conduction gel and agarose gel to stimulate the worm. The worms were placed on an agarose gel with a small hole (diameter = 2 mm, thickness = 2 mm), which was filled with 10 μL M9 buffer. The distance between the worm (the bottom of the hole) and the FUS transducer is 2 mm, allowing the worm to lie within the focal zone of US. (B) Expression of Ppkd2-Venus-MM-Prestin(N548S, V715G) in ray neurons located in the fan structure of adult male tails. Scale bar: 20 μm. (C) Time-lapse images showing tail curling of *Ppkd2-Venus-MM-Prestin(N548S, V715G)* transgenic worms induced by FUS stimulation. Arrowheads indicate the curling response. Scale bar: 50 μm. (D) FUS at 500 kPa peak negative pressure triggered ventral tail curling through activation of ray sensory neurons expressing Venus-MM-Prestin(N548S, V715G). All males carried him-5(e1409) mutation to increase the male population. "WT" denotes wild-type gene expression in all Ppkd2-expressing cells (CEMs, HOB, and eight pairs of ray sensory neurons). Curling frequency was defined as the number of curling responses divided by the number of trials. Data (red) represent mean ± S.E.M. from 10 worms per group. Student's *t*-test was used to determine statistical significance. (E) FUS at 500 kPa did not affect worm survival, regardless of the expression of Venus-MM-Prestin(N548S, V715G). Survival was assessed two days post-treatment from 10 worms per group. (F) Demonstration of the setup used to transmit FUS wave forms into the intact mouse brain and record the tail movement. (G) Representative images of motor cortex brain sections with or without FUS stimulation. Neuronal activity was assessed via immunostaining of c-Fos (red) in neurons expression Venus or Venus-MM-Prestin(N548S, V715G) following FUS stimulation (0.5 MHz, 0.5 MPa, 10 Hz PRF, 2000 cycles per burst, 5-min duration). Asterisks indicate c-Fos⁺/Venus⁺ double-positive neurons. Scale bar: 100 μm. (H) Quantification of the percentage of c-Fos-positive neurons among Venus-expressing cells, with or without FUS stimulation. Data (red) represent mean ± S.E.M. from 10-12 sections, derived from 3 mice per condition. Student's *t*-test was used to determine statistical significance. (I) Quantification of the tail oscillation range among Venus alone or Venus-MM-Prestin(N548S, V715G) mice, with or without FUS stimulation. Tail movement was quantified as the difference in movement distance before and during FUS stimulation. Data (red) represent mean ± S.E.M. from 2-3 mice per group. Student's *t*-test was used to assess statistical significance.

We further investigated the potential of MM-Prestin(N548S, V715G) to modulate animal behavior. Ray sensory neurons located in the tails of male *C. elegans* are responsible for sensing contact during mating. Activation of these neurons induces ventral curling of the tail 37. To achieve targeted expression, we generated *Ppkd2-Venus-MM-Prestin(N548S, V715G)* transgenic worms. Expression of Venus-MM-Prestin(N548S, V715G) in ray sensory neurons, driven by the tissue-specific pkd-2 promoter, was confirmed by live-cell imaging (Figure 5B). The transgenic worms were placed on agarose gel in direct contact with an US transducer and coupling cones. Their behavior was monitored in real-time under a dissecting microscope during FUS stimulation (Figure 5A). Quantitative analysis revealed that FUS stimulation at acoustic pressures above 500 kPa significantly induced ventral bending of the male tail (Figure 5C, D). Importantly, this stimulation paradigm was well tolerated, as it did not affect the worms' morphology, locomotion and lifespan (Figure 5E). We also tested whether MM-Prestin(N548S, V715G) can be used to activate the motor cortex and elicit mouse tail movements (Figure 5F)—an established behavioral readout in previous studies 42,43. Our results show that FUS stimulation robustly activates motor-cortex neurons expressing Venus-MM-Prestin(N548S, V715G), but not those in the control group (Figure 5G, H). Moreover, MM-Prestin(N548S, V715G)-mediated neuromodulation reliably evoked tail movements in mice (Figure 5I). Taken together, these results demonstrate that MM-Prestin(N548S, V715G) enables US-mediated neuromodulation across species and tissue types.

### Safety Assessment of *In Vivo* Sonogenetic Stimulation

**Figure 6:**
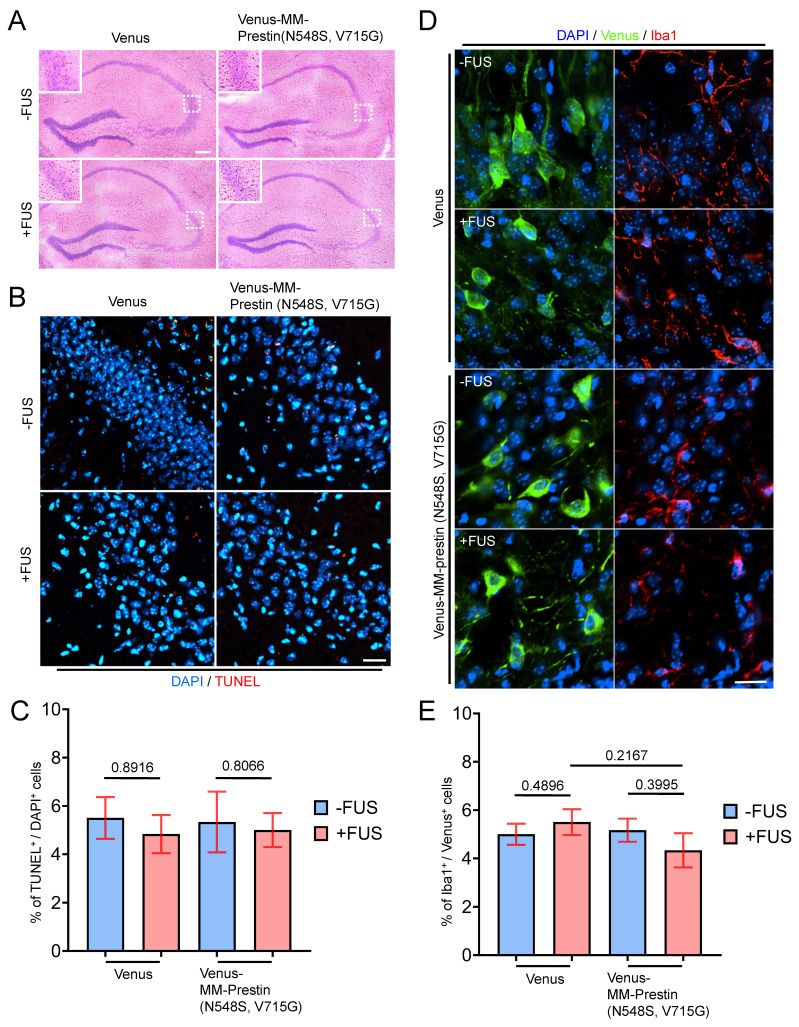
Safety evaluation of FUS effects on brain tissue. (A) Hematoxylin and eosin (H&E) staining of brain sections showing erythrocyte extravasation in the hippocampal region following the indicated treatments (FUS parameter: 0.5 MHz, 0.5 MPa, 10 Hz PRF, 2000 cycles per burst, 5-min duration). Scale bar: 100 μm. (B) TUNEL staining was performed to assess apoptotic cell death in the hippocampus. Scale bar: 20 μm. (C) Quantification of TUNEL-positive cells indicating apoptosis. Data are presented as mean ± SD; n = 3 mice per group. (D) Immunohistochemical images showing microglial activation in the indicated regions of interest (ROIs), as detected by Iba1 antibody (red fluorescence). Scale bar: 10 μm. (E) Quantification of Iba and Venus double positive cells indicating the activation of microglia. Data are presented as mean ± SD; n = 3 mice per group.

To verify the safety of our *in vivo* sonogenetic stimulation, we evaluated potential histological and immune responses in brain tissue. Hematoxylin and eosin (H&E) staining was performed to assess structural integrity and erythrocyte leakage, while TUNEL assays were used to detect apoptotic cells in the hippocampus. No tissue disruption or hemorrhage was observed (Figure 6A), and no significant differences in apoptotic cell counts (Figure 6B, C) were observed in all groups, including Venus-only, Venus+FUS, Venus-MM-Prestin(N548S, V715G), and Venus-MM-Prestin(N548S, V715G)+FUS, suggesting that the applied US parameters did not cause overt neuronal damage. Besides, a microglial marker, Iba1, was used to examine potential immune activation. No significant differences in Iba1 signal were observed among the groups, including those that received AAV-Venus or AAV-Venus-MM-Prestin(N548S, V715G) with or without FUS stimulation (Figure 6D, E). These results indicate that AAV delivery and the sonogenetic protocol used in our study did not trigger substantial immune activation or cytotoxic effects in the brain. Together, these findings confirm that MM-Prestin(N548S, V715G) confers effective and safe US sensitivity to targeted neurons in the mouse brain.

## Discussion

In this study, we systematically characterized the US responsiveness of Prestins derived from echolocating bats and engineered mouse Prestin variants, ultimately identifying a novel mutant—MM-Prestin(N548S, V715G)—that enables robust and specific sonogenetic control of neural activity and animal behavior. While bat-derived Prestins were hypothesized to exhibit high US sensitivity due to their evolutionary adaptations, our functional assays revealed that they neither formed clusters nor triggered US-induced calcium responses in human HEK293T cells (Figure S1; Figure S2) and primary neurons (Figure S3). In contrast, a mutagenesis screen uncovered MM-Prestin(N548S, V715G) as a potent US-responsive variant with enhanced clustering and strong calcium influx in response to pulsed US (Figure 1; Figure 2). This enhanced functionality extended beyond cell lines to primary cortical neurons, mouse brains, and *C. elegans*, demonstrating that MM-Prestin(N548S, V715G) can serve as an effective sonogenetic actuator across multiple biological systems (Figure 3; Figure 4; Figure 5). These findings establish a powerful platform for non-invasive modulation of cellular activity and behavior through targeted US stimulation.

Our results found that prestin from four echolocating bats did not confer US sensitivity in human HEK293T cells (Figure S2), suggesting that N7T and N308S mutations only enhance US sensitivity of mouse Prestin but not that of the four echolocating bats Prestins. Moreover, 0.5 MHz is sufficient to stimulate human and mouse cells expressing MM-Prestin(N7T, N308S); whereas 80 kHz, a frequency close to the bat US hearing range, cannot 28. This observation also implies that the mechanism by which MM-Prestin(N7T, N308S) responds to FUS is different from that of US hearing mediated by bat Prestins. The high correlation between puncta formation and the US sensitivity of Prestin variants encouraged us to boost Prestin clustering. After screening, we found both N548S and V715G mutations are important to confer better protein clustering and US sensitivity of MM-Prestin. Based on protein structural prediction, all Prestin variants tested in this study shared similar structures (Figure S6), assuming different protein clustering of these variants is unlikely determined by their protein structures. AlphaFold-based structural prediction of the prestin dimer indicates that N548S and V715G are not positioned within the canonical interaction interface responsible for dimer formation. This observation suggests that these mutations likely enhance oligomerization and US sensitivity through alternative mechanisms, rather than directly modifying the dimerization interface. Previous studies have shown that prestin dimer/oligomer formation can be influenced by STAS (Sulfate transporter and Anti-Sigma factor antagonist domain)-domain stability, membrane packing, and conformational transitions associated with electromotility 44-46. These factors offer plausible explanations for how N548S and V715G may indirectly modulate clustering behavior and US responsiveness, even without residing in the dimer interface. Notably, both N548S and V715G are located within the STAS domain, a highly conserved and cytosolic domain at the C-terminus of Prestin 47. It has been reported that STAS domain regulates the intracellular protein-protein interaction and may influence Prestin's responsiveness to anions such as chloride, which are crucial for its voltage-sensing mechanism. Whether N548S and V715G mutations affect the properties of STAS domain is worth exploring in the future. Furthermore, the weak clustering and lack of US responsiveness of bat Prestins in HEK293T cells and primary neurons are noteworthy. As previous studies have shown that Prestin function can be strongly influenced by the cellular context and supporting molecular machinery 39,46,47, we assumed that bat Prestins may require species-specific membrane environments, lipid compositions, post-translational modifications, or interacting proteins that are not present in the heterologous systems used here. Further investigation in bat-derived or sensory epithelial cell types will be valuable for fully understanding the functional requirements of bat Prestins.

A short-pulsed FUS induces a sustained calcium response in cells expressing MM-Prestin(N7T, N308S) or MM-Prestin(N548S, V715G) (Figure 2; Figure 3), likely due to the continued vibration of Prestin puncta after FUS cessation (Figure 1H) 28. Although prolonged calcium elevation may theoretically reduce the temporal precision of inducible neuromodulation, extended intracellular Ca²⁺ signals are well known to increase the probability and robustness of downstream neuromodulatory effects. For instance, in optogenetics, OptoSTIM1 activation produces sustained Ca²⁺ influx lasting minutes, thereby strongly engaging CaMKII, CREB, and NFAT pathways and promoting long-term neuronal modulation 48. Likewise, Gq-based optogenetic tools such as Opto-α1AR generate long-lasting Ca²⁺ plateaus (5-20 min) that more effectively drive neuromodulatory and behavioral responses, even when triggered by brief light pulses 49. These studies collectively demonstrate that extended Ca²⁺ transients can amplify neuromodulatory efficacy, particularly in applications where sustained signaling is beneficial. Importantly, long-lasting calcium elevations produced by these optogenetic systems did not lead to cellular toxicity or neuronal injury 48,49. Consistent with these findings, MM-Prestin(N548S, V715G)-evoked calcium responses did not impair the viability of cultured cells (Figure S5) or *C. elegans* (Figure 5E) and did not cause tissue damage in mouse brains (Figure 6). Leveraging these prolonged Ca²⁺ dynamics, we successfully applied Prestin-mediated sonogenetics to chronically attenuate epileptic activity and ameliorate motor symptoms of Parkinson's disease *in vivo* 6,8. Based on the superior US sensitivity of MM-Prestin(N548S, V715G), this variant holds strong potential for broader applications in neuromodulation and other sonogenetics-based therapeutic strategies in the future.

Accumulating studies have identified several classes of US-responsive proteins, including mechanosensitive ion channels and gas vesicles 5. However, many mechanosensitive channels (e.g., Piezo1 and members of the TRP family) are ubiquitously expressed across diverse tissues 50,51, making them susceptible to unintended activation in non-target cells and contributing to confounding off-target or peripheral auditory effects. In addition, the large gene size of Piezo1 and gas vesicle gene clusters presents practical challenges for efficient viral packaging and delivery 11,12,52. In contrast, Prestin offers several distinct advantages. Its endogenous expression is highly restricted to cochlear outer hair cells 53, providing excellent orthogonality when ectopically expressed in other tissues. Moreover, the FUS parameters required to activate Prestin do not trigger auditory pathway activation 28, thereby minimizing undesired sensory or off-target responses. Prestin also possesses a compact gene size that is readily accommodated by standard viral vectors, enabling robust delivery across species and tissues (Figure 4; Figure 5) 6,8,28. Together, these properties make Prestin a convenient, selective, and broadly applicable sonogenetic actuator.

## Supplementary Material

Supplementary figures and information.

## Abbreviations

US: ultrasound
FUS: focused ultrasound
AP: anteroposterior
ML: mediolateral
DV: dorsoventral
AAV: adeno-associated virus
PRF: pulse repetition frequency
IHC: Immunohistochemistry
H&E: hematoxylin and eosin
TUNEL: terminal deoxynucleotidyl transferase biotin-dUTP nick end labeling
BSA: bovine serum albumin
CB: Chicken β-actin promoter
CMV: cytomegalovirus promoter
CREB: cAMP response element-binding protein
DAPI: 4′,6-diamidino-2-phenylindole
FRET: Förster resonance energy transfer
