# Supplementary material for: Enhancing prestin oligomerization for superior sonogenetics
Source: Theranostics. 2026 Jan 1;16(7):3321–37. doi: 10.7150/thno.122468 (PMC12846731; doi:10.7150/thno.122468)
Supplement: Supplementary file 1 — Supplementary figures and information. [file thnov16p3321s1.pdf]

SUPPLEMENTARY MATERIALS

Enhancing prestin oligomerization for superior sonogenetics

Hsien-Chu Wang[1#], Thi-Nhan Phan[2#], Tzu-Han Chen[1#], Yun Lin[1], Ning Hsu[1], Ching-Hsiang Fan[3,4,5], Po-Han Chiang[6,7], Chun-Hao Chen[8], Chih-Kuang Yeh[2,9,10]*, Yu-Chun Lin[1,11]*

[1]Institute of Molecular Medicine, National Tsing Hua University, Hsinchu 300044, Taiwan.

[2]Department of Biomedical Engineering and Environmental Sciences, National Tsing Hua University, Hsinchu 300044, Taiwan.

[3]Department of Biomedical Engineering, National Cheng Kung University, Tainan 701401, Taiwan.

[4]Medical Device Innovation Center, National Cheng Kung University, Tainan 701401, Taiwan.

[5]Center of Transformative Bioelectronic Medicine, College of Medicine, National Cheng Kung University, Tainan 701401, Taiwan.

[6]Institute of Intelligent Bioelectrical Engineering, National Yang Ming Chiao Tung University, Hsinchu 300093, Taiwan.

[7]Department of Electronics and Electrical Engineering, National Yang Ming Chiao Tung University, Hsinchu 300093, Taiwan.

[8]Institute of Molecular and Cellular Biology, National Taiwan University, Taipei 106319, Taiwan.

[9]Department of Biomedical Sciences and Engineering, Tzu Chi University, Hualien 970374, Taiwan.

[10]Department of Biomedical Engineering, Chung Yuan Christian University, Taoyuan 320314, Taiwan.

[11]Department of Medical Science, National Tsing Hua University, Hsinchu 300044, Taiwan.

# These authors contribute equally; *Corresponding author: Yu-Chun Lin (ycl@life.nthu.edu.tw); Chih-Kuang Yeh (ckyeh@mx.nthu.edu.tw)

Table of Contents

Figure

Figure S1. Puncta formation of prestins from different species.

Figure S2. Prestins from four echolocating bats do not confer US sensitivity to cells.

Figure S3. Prestins from four echolocating bats do not, or only weakly, confer utrasound sensitivity to primary neurons.

Figure S4. Representative images of 192 Venus-tagged MM-Prestin variants generated by random mutagenesis.

Figure S5. Assessment of cell viability following FUS stimulation using CCK-8 assay.

Figure S6. Alignment of different prestin variants.

Protein sequence of prestin variants

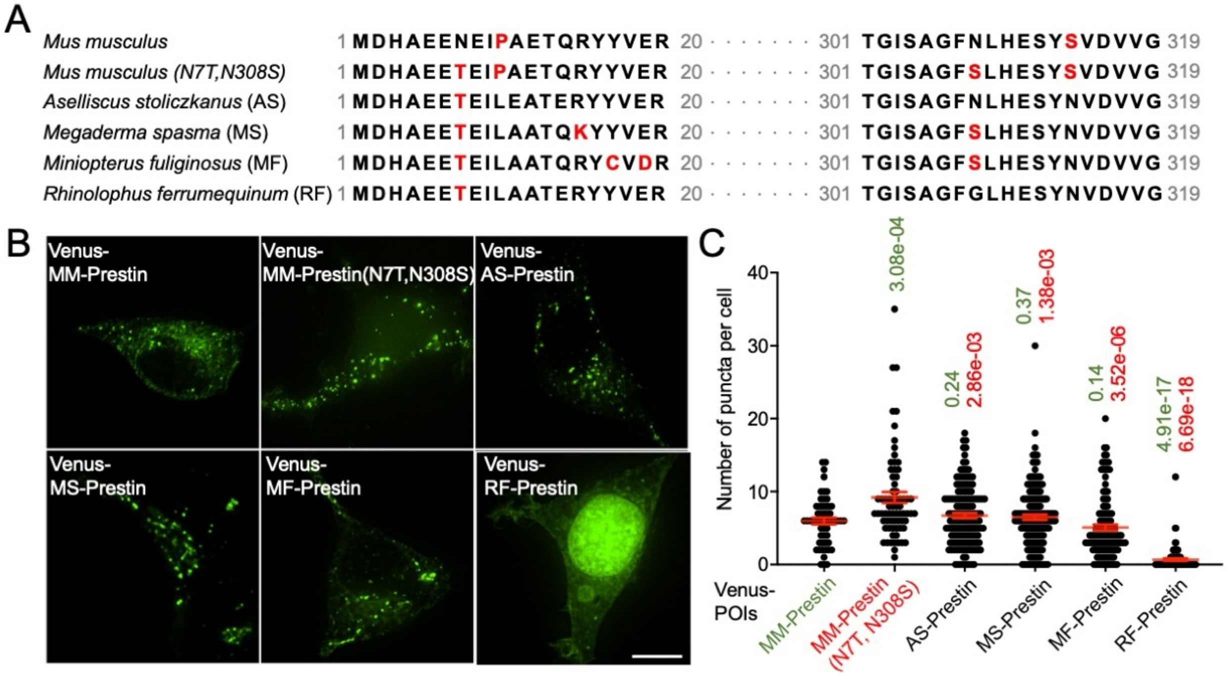

Figure S1. Puncta formation of prestins from different species. (A) Sequence alignment of prestins from four echolocating bats (*Aselliscus stoliczkanus*, *Megaderma spasma, Miniopterus fuliginosus, Rhinolophus ferrumequinum*) and mouse (Mus musculus), with or without the N7T and N308S mutations. Residues highlighted in red indicate amino acids that diverge from the conserved sequence shared by most species. (B) Representative fluorescence images of HEK293T cells transfected with the indicated constructs: Venus-MM-Prestin, Venus-MM-Prestin(N7T, N308S), Venus-AS-Prestin, Venus-MS-Prestin, Venus-MF-Prestin, and Venus-RF-Prestin. Maximum intensity z-projections were generated from 17 stacks, each 0.3 μm apart. Scale bar, 10 μm. (C) Quantification of puncta number for the indicated constructs shown in B. Data (in red) represent mean ± S.E.M. from at least three independent experiments; n = 55, 70, 141, 147, 118, and 90 cells (from left to right). Student's *t*-test was used to compare values between the indicated construct and MM-Prestin (green) or MM-Prestin(N7T, N308S) (red), respectively.

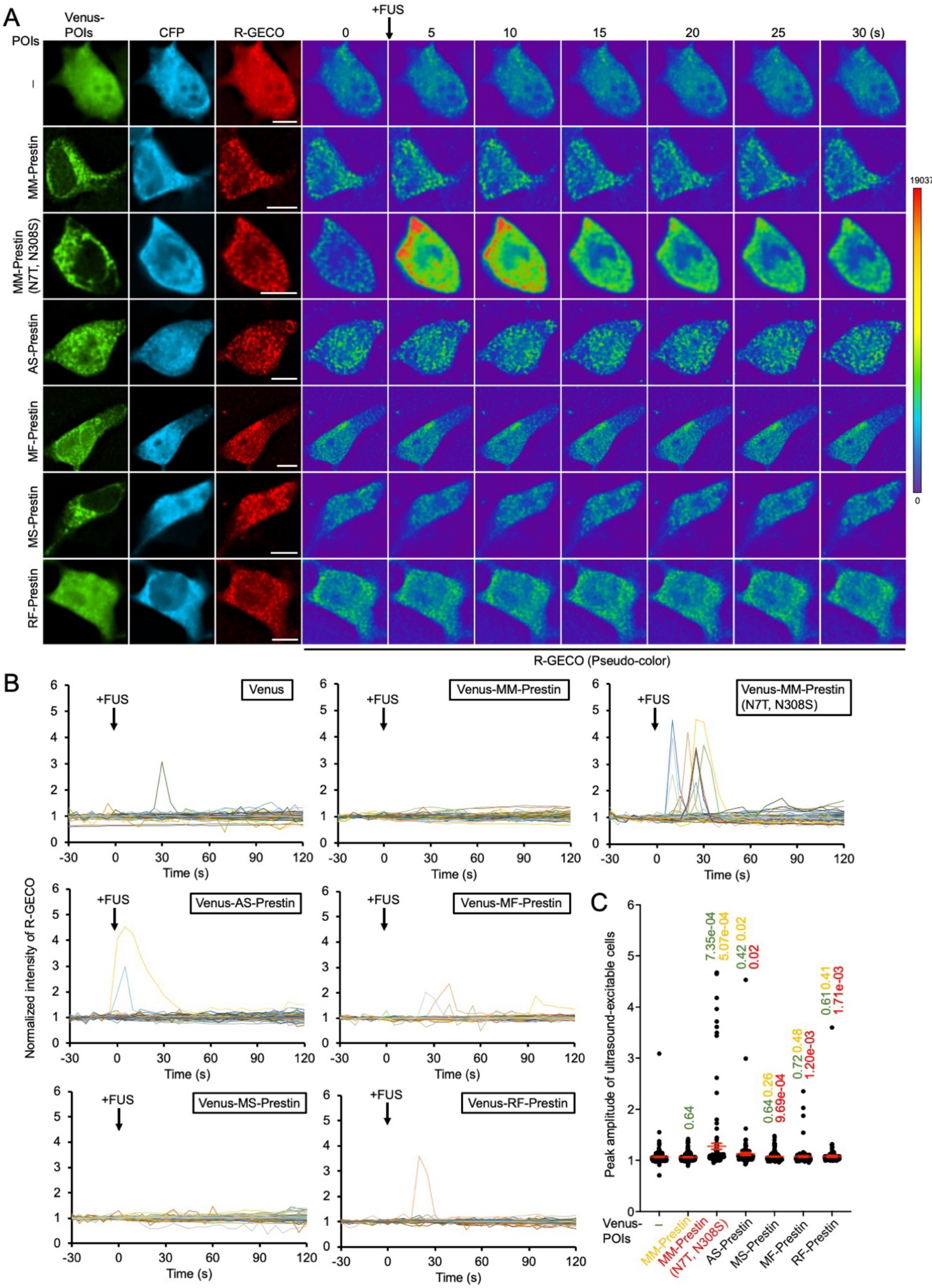

A
Venus-POIs
CFP
R-GECO
+FUS
0
5
10
15
20
25
30 (s)
POIs
–
MM-Prestin
MM-Prestin (N7T, N308S)
AS-Prestin
MF-Prestin
MS-Prestin
RF-Prestin
19037
0
R-GECO (Pseudo-color)
B
Venus
Venus-MM-Prestin
Venus-MM-Prestin (N7T, N308S)
Venus-AS-Prestin
Venus-MF-Prestin
Venus-MS-Prestin
Venus-RF-Prestin
Normalized intensity of R-GECO
Time (s)
C
Peak amplitude of ultrasound-excitable cells
Venus-POIs
0.64
7.35e-04
5.07e-04
0.42
0.02
0.02
0.64
0.26
9.69e-04
0.72
0.48
1.20e-03
0.61
0.41
1.71e-03

Figure S2. Prestins from four echolocating bats do not confer US sensitivity to cells. (A) HEK293T cells were co-transfected with CFP-R-GECO and the indicated constructs. Twenty-four hours post-transfection, a pulse of FUS (0.5 MHz, 0.5 MPa, 10 Hz PRF, 2000 cycles, 3-sec duration) was applied, and R-GECO fluorescence intensity was monitored in real time (shown in pseudo-color). Scale bar: 10 μm. (B) Time course of normalized R-GECO fluorescence intensity in cells expressing the indicated constructs following 0.5 MHz FUS stimulation. Data were collected from at least three independent experiments; n = 255, 210, 140, 153, 136, 138, and 152 cells for Venus, Venus-MM-Prestin, Venus-MM-Prestin(N7T, N308S), Venus-AS-Prestin, Venus-MF-Prestin, Venus-MS-Prestin, and Venus-RF-Prestin groups, respectively. (C) Peak R-GECO fluorescence amplitude in response to FUS stimulation in cells expressing the indicated constructs. Data (in red) represent mean ± S.E.M. from more than three independent experiments. Student's *t*-test was used to compare values with Venus alone (green), MM-Prestin (orange), or MM-Prestin(N7T, N308S) (red), respectively.

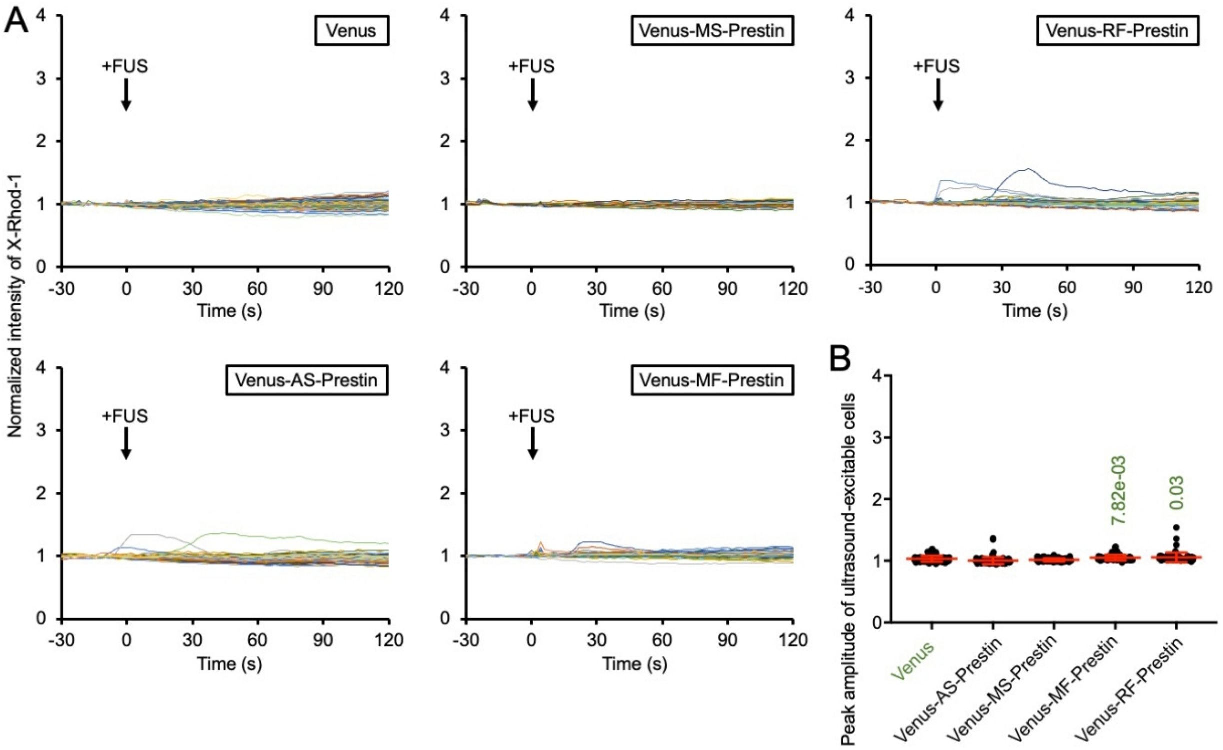

Figure S3. Prestins from four echolocating bats do not, or only weakly, confer US sensitivity to primary neurons. (A) Rat primary cortical neurons were transfected with plasmids encoding either Venus or Venus-tagged Prestins from four echolocating bat species. Transfected neurons were stained with the calcium indicator X-Rhod-1. Time course of X-Rhod-1 fluorescence intensity following FUS stimulation (0.5 MHz, 0.5 MPa, 10 Hz PRF, 2000 cycles, 3-sec duration) was monitored by live-cell imaging. Data were collected from three independent experiments: n = 129, 112, 88, 79, and 75 cells for the Venus, Venus-AS-Prestin, Venus-MS-Prestin, Venus-MF-Prestin, and Venus-RF-Prestin groups, respectively. (B) Peak X-Rhod-1 fluorescence intensity quantified from A. Data (in red) present mean ± S.E.M. Student's *t*-test was used to compare Venus with each indicated conditions.

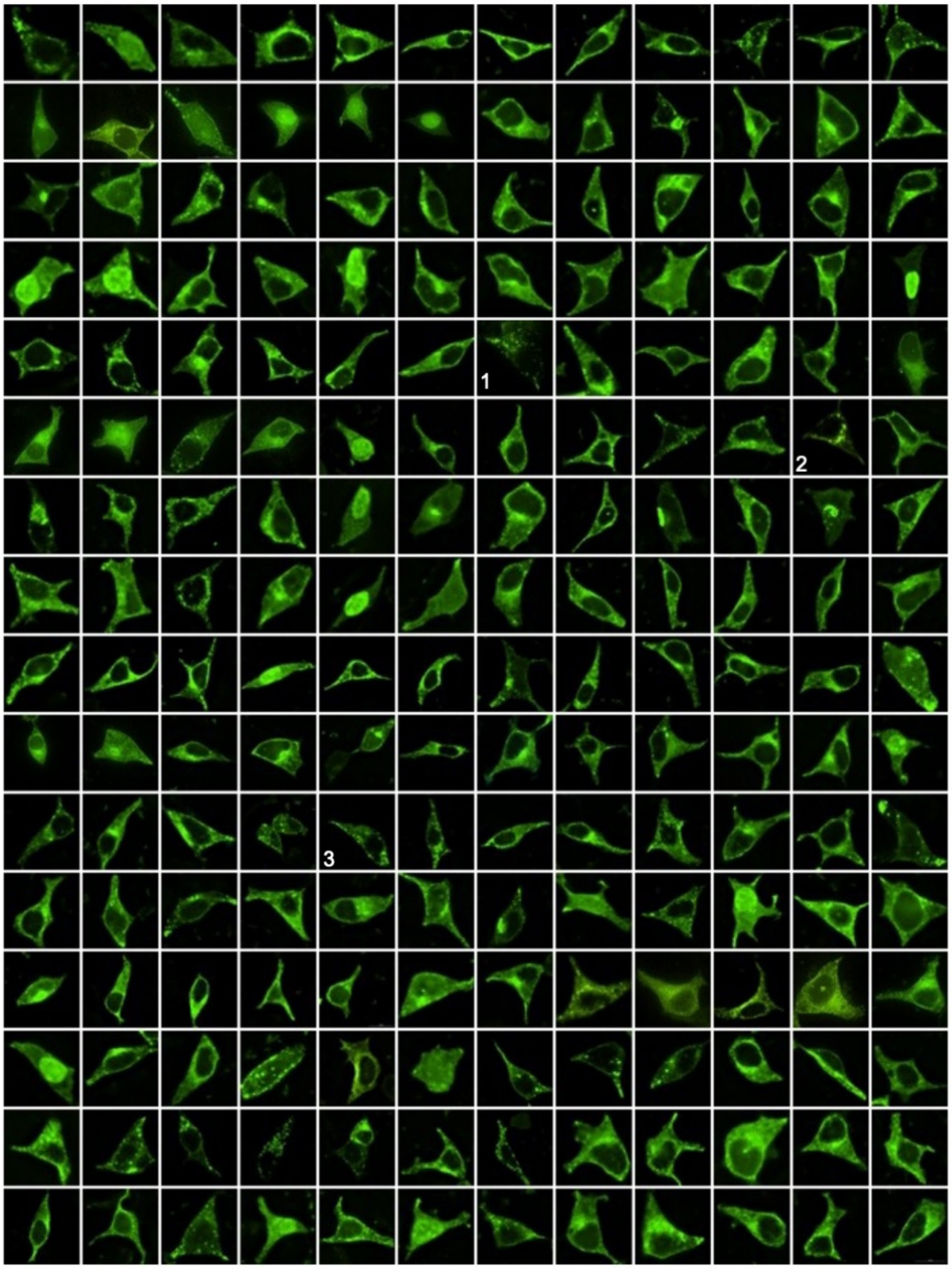

1. MM-Prestin(N548S, V715G)
2. MM-Prestin(V79H, F200V, F241L, L484S, R502G, K557E)
3. MM-Prestin(N7T, N308S, Q630R)

Figure S4. Representative images of 192 Venus-tagged MM-Prestin variants generated by random mutagenesis. HEK293T cells were transfected with Venus-tagged MM-Prestin variants. Several variants exhibiting strong protein clustering were selected for sequencing. Scale bar, 10 μm.

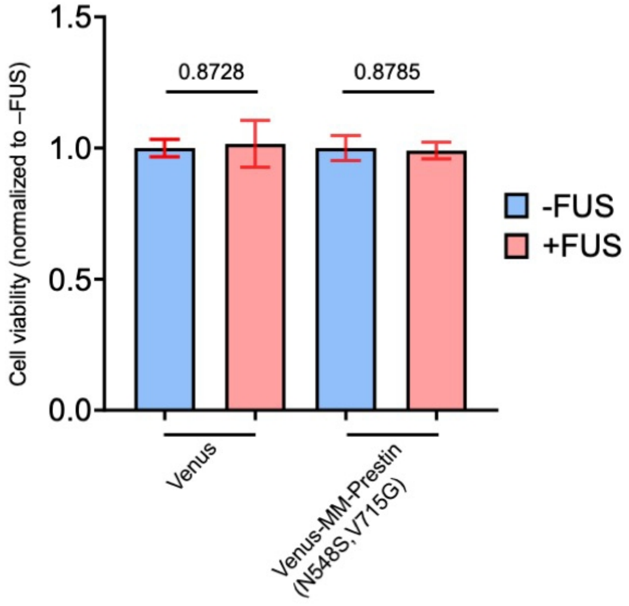

Figure S5. Assessment of cell viability following FUS stimulation using CCK-8 assay. HEK293T cells expressing either Venus or Venus-MM-Prestin(N548S, V715G) were exposed to FUS (0.5 MHz, 0.5 MPa, 10 Hz PRF, 2000 cycles, 3-s duration). Cell viability was assessed 3 hours post-stimulation using the CCK-8 assay, with absorbance at 450 nm. Data represent mean ± S.E.M. from three independent experiments.

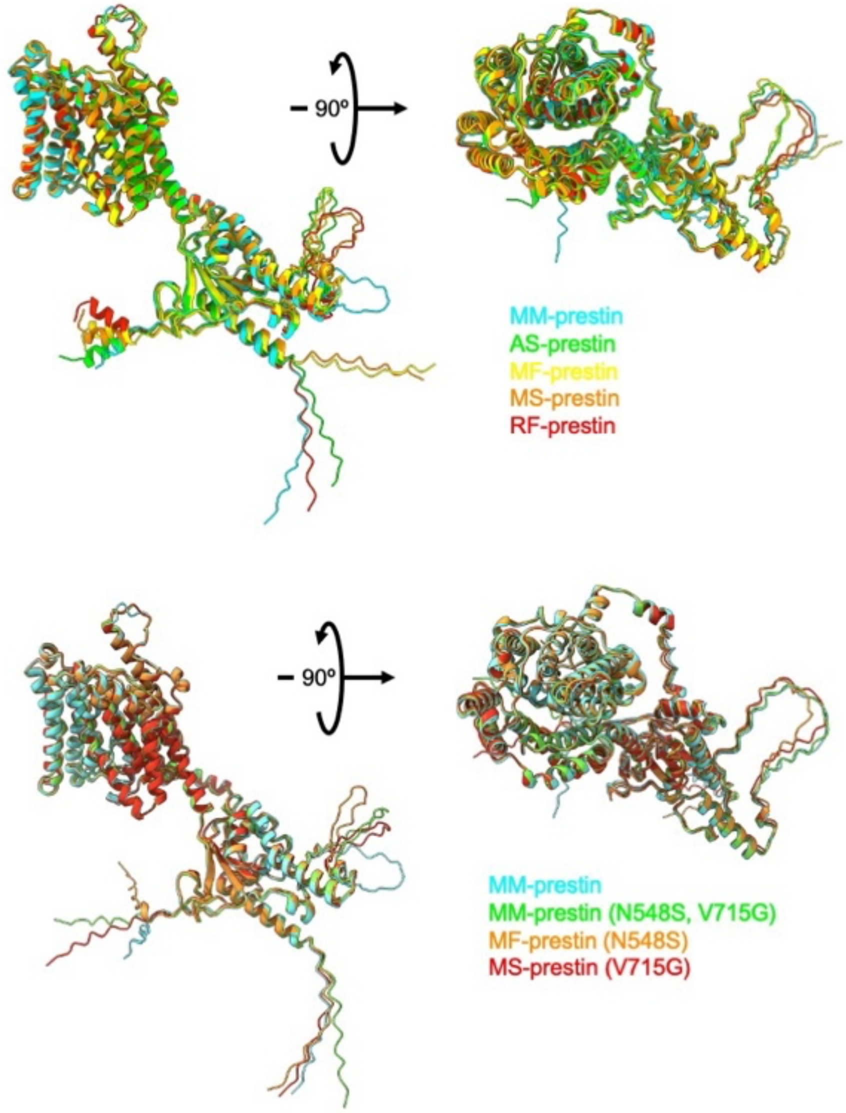

Figure S6. Alignment of different prestin variants. Protein structures of the indicated prestin variants were predicted using AlphaFold and aligned using ChimeraX software.

Protein sequence of different prestin variants

MM-Prestin

MDHAEENEIPAETQRYYVERPIFSHPVLQERLHVKDKVTESIGDKLKQAFTCTPK
KIRNIIYMFLPITKWLPAYKFKEYVLGDLVSGISTGVLQLPQGLAFAMLAAVPPVF
GLYSSFYPVIMYCFFGTSRHISIGPFAVISLMIGGVAVRLVPDDIVIPGGVNATNGTE
ARDALRVKVAMSVTLLSGIIQFCLGVCRFGFVAIYLTEPLVRGFTTAAAVHVFTSM
LKYLFGVKTKRYSGIFSVVYSTVAVLQNVKNLNVCSLGVGLMVFGLLLGGKEFN
ERFKEKLPAPIPLEFFAVVMGTGISAGFNLHESYSVDVVGTLPLGLLPPANPDTSLF
HLVYVDAIAIAIVGFSVTISMAKTLANKHGYQVDGNQELIALGICNSIGSLFQTFSI
SCSLSRSLVQEGTGGKTQLAGCLASLMILLVILATGFLFESLPQAVLSAIVIVNLKG
MFMQFSDLPFFWRTSKIELTIWLTTFVSSLFLGLDYGLITAVIIALLTVIYRTQSPSY
KVLGQLPDTDVYIDIDAYEEVKEIPGIKIFQINAPIYYANSDLYSSALKRKTGVNPA
LIMGARRKAMRKYAKEVGNANVANATVVKVDAEVDGENATKPEEEDDEVKFPP
IVIKTTFPEELQRFLPQGENVHTVILDFTQVNFVDSVGVKTLAGIVKEYGDVGIYV
YLAGCSPQVVNDLTRNNFFENPALKELLFHSIHDAVLGSQVREAMAEQEATASLP
QEDMEPNATPTTPEA

MM-Prestin(N7T, N308S)

MDHAEETEIPAETQRYYVERPIFSHPVLQERLHVKDKVTESIGDKLKQAFTCTPK
KIRNIIYMFLPITKWLPAYKFKEYVLGDLVSGISTGVLQLPQGLAFAMLAAVPPVF
GLYSSFYPVIMYCFFGTSRHISIGPFAVISLMIGGVAVRLVPDDIVIPGGVNATNGTE
ARDALRVKVAMSVTLLSGIIQFCLGVCRFGFVAIYLTEPLVRGFTTAAAVHVFTSM
LKYLFGVKTKRYSGIFSVVYSTVAVLQNVKNLNVCSLGVGLMVFGLLLGGKEFN
ERFKEKLPAPIPLEFFAVVMGTGISAGFSLHESYSVDVVGTLPLGLLPPANPDTSLF
HLVYVDAIAIAIVGFSVTISMAKTLANKHGYQVDGNQELIALGICNSIGSLFQTFSI
SCSLSRSLVQEGTGGKTQLAGCLASLMILLVILATGFLFESLPQAVLSAIVIVNLKG
MFMQFSDLPFFWRTSKIELTIWLTTFVSSLFLGLDYGLITAVIIALLTVIYRTQSPSY
KVLGQLPDTDVYIDIDAYEEVKEIPGIKIFQINAPIYYANSDLYSSALKRKTGVNPA
LIMGARRKAMRKYAKEVGNANVANATVVKVDAEVDGENATKPEEEDDEVKFPP
IVIKTTFPEELQRFLPQGENVHTVILDFTQVNFVDSVGVKTLAGIVKEYGDVGIYV
YLAGCSPQVVNDLTRNNFFENPALKELLFHSIHDAVLGSQVREAMAEQEATASLP
QEDMEPNATPTTPEA

AS-Prestin

MDHAEETEILEATERYYVERPIFSHLALQERLHKKDKISDSIGDKLKQAFTCTPKK
IRNIIYMFLPITKWLPAYNFKEYVLGDLVSGVSTGVLQLPQGLAFAMLAAVPPVFG
LYSSFYPVIMYCFFGTSRHISIGPFAVISLMIGGVAVRLVPDDIAPPGGVNATNSTEF
RDALRVKVAMSVTLLAGIIQFCLGVCRFGFVAIYLTEPLVRGFTTAAAVHVATSML
KYLFGVKTKRYSGIFSVVYSTVAVLQNVKNLNVCSLGVGLMVFGLLLGGKEFNE
RFKEKLPAPIPLEFFAVVMGTGISAGFNLHESYNVDVVGTLPLGLLPPANPDTSLF
HLVYVDAIAIAIVGFSVTISMAKSLANKHGYQVDGNQELIALGLCNSTGSLFQTFA
ISCSLSRSLVQEGTGGKTQLAGCLASLMILMVILATGFLFESLPQAVLSAIVIVNLK
GMFMQFSDLPFFWRTSKIELTIWLSTFVSSLFLGLDYGLITAVIIALMTVIYRTQSPT
YTVLGQLPDTDVYIDKDAYEEVKEIPGIKIFQINAPIYYANSDLYSSALKTKTGVNP
SFILGARRKAIKKYAKEGGNINIANAVDVKADAEVDAEDGTKPEEEEEEIKYPPIV
TKSTLPEELQRFMPPLENVHTVILDFTQVNFIDSVGVKTLAGIVKEYGDVGIYVYL
AGCSAQVVSDLTRNQFFENPALLELLFHSIHDAVLGSLVREALEEKEAAATTPQED
SEPNATPEV

MS-Prestin
MDHAEETEILAATQKYYVERPIFSHSVLQERLHKKDKISDSIGDKLKQAFTCTPKK
IRNIIYMFLPITKWLPAYNFKEYVLGDLVSGISTGVLQLPQGLAFAMLAAVPPVFG
LYSSFYPVIMYCFFGTSRHISIGPFAVISLMIGGVAVRLVPDDIVIPGGVNATNGTEA
RDALRVKVAMSVTLLSGIIQFCLGVCRFGFVAIYLTEPLVRGFTTAAAVHVFTSML
KYLFGVKTKRYSGIFSVVYSTVAVLQNVKNLNVCSLGVGLMVFGLLLGGKEFNE
RFKEKLPAPIPLEFFAVVMGTGISAGFSLHESYNVDVVGTLPLGLLPPANPDTSLFH
LVYVDAIAIAIVGFSVTISMAKTLANKHGYQVDGNQELIALGLCNSIGSLFQTFSIS
CSLSRSLVQEGTGGKTQLAGCLASLMILMVILATGFLFESLPQAVLSAIVIVNLKG
MFMQFSDLPFFWRTSKIELTIWLTTFVSSLFLGLDYGLITAVIIALMTVIYRTQSPSY
KVLGQLPDTDVYIDIDAYEEVKEIPGVKIFQINAPIYYANSDLYSSALKRKTGVNP
TLIMGARRKAMKKYAKEVGNANMVNATVVKADAEVDGGDGTKPEEEDDEIKF
PPIVTKSTFPEELQRFMPPGDNIHTVILDFTQVNFIDSVGVKTLSGIVKEYGDVGIY
VYLAGCSPQVVNDLTQNQFFENPALLELLFHSIHDAVLGSQVREALAEQEALAPP
PQEDSEPNATPEP

MF-Prestin
MDHAEETEILAATQRYCVDRPLFSHLALQERLHKKDKIPDSIGDKLKQAFTCTPK
KIRNIIYMFLPITKWLPAYKFKEYVLGDLVSGISTGVLQLPQGLAFAMLAAVPPVF
GLYSSFYPVIMYCFFGTSRHISIGPFAVISLMIGGVAVRLVPDDIVIPGGVNATNGTEI

RDALRVKVAMSVTLLSGIIQFCLGVCRFGFVAIYLTEPLVRGFTTAAAVHVFTSML
KYLFGVKTKRYSGIFSVVYSTVAVLQNVKNLNVCSLGVGLMVFGLLLGGKEFNE
RFKEKLPAPIPLEFFAVVMGTGISAGFSLHESYNVDVVGTLPLGLLPPANPDTSLFH
LVYVDAIAIAIVGFSVTISMAKTLANKHGYQVDGNQELIALGLCNSIGSLFQTFSIS
CSLSRSLVQEGTGGKTQLAGCLASLMILMVILATGFLFESLPQAVLSAIVIVNLKG
MFMQFSDLPFFWRTSKIELTIWLTTFVSSLFLGLDYGLITAVIIALMTVIYRTQSPSY
KVLGQLPDTDVYIDIDAYEEVKEIPGIKIFQVNAPIYYANSDLYSSALKRKTGVNP
ALIMGARRKAMKKYAKEVGNANMANATIVKADGEVDGEDATKPEEDDEEIKFP
PIVIKSTFPEELQRFMPPGDNVHTVILDFTQVNFIDSVGVKTLSGIVKEYGDVGIY
VYLAGCSAQVVNDLTQNLFFENPALKELLFHSIHDAVLGSQLREALAEQEALTPPP
QEDSEPNATPEA

RF-Prestin
MDHAEETEILAATERYYVERPIFSHLVLQERLHKKDKISDSIGDKLKQAFTCTPKK
IRNIIYMFLPITKWLPAYNFKEYVLGDLVSGISTGVLQLPQGLAFAMLAAVPPVFG
LYSSFYPVIMYCFFGTSRHISIGPFAVISLMIGGVAVRLVPDDIAVPGGVNATNGTEF
RDALRVKVAMSVTLLAGIIQFCLGVCRFGFVAIYLTEPLVRGFTTAAAVHVFTSML
KYLFGVKTKRYSGIFSVVYSTVAVLQNVKNLNVCSLGVGLMVFGLLLGGKEFNE
RFKEKLPAPIPLEFFAVVMGTGISAGFGLHESYNVDVVGTLPLGLLPPANPDTSLF
HLVYVDAIAIAIVGFSVTISMAKTLANKHGYQVDGNQELIALGLCNSTGSLFQTF
AISCSLSRSLVQEGIGGKTQLAGCLASLMILMVILATGFLFESLPQAVLSAIVIVNL
KGMFMQFSDLPFFWRTSKIELVIWLSTFVSSLFLGLDYGLITAVIIALMTVIYRTQS
PTYTVLGQLPDTDVYIDIDAYEEVKEIPGIKIFQINAPIYYANSDLYSNALKRKTGV
NPSFILGARRKAMKKYAKEGGNINIANATDVKADAEVDAEDGTKPEEEEDEVKY
PPVVIKSTFPEELQRFMPPLENVHTIILDFTQVNFIDSVGVKTLQGIVKEYGDVGIY
VYLAGCSAQVVSDLTRNRFFENPALLDLLFHSIHDAVLGSLVREALEEKEAAAAT
PQEDSEPNATPDV

MM-Prestin(N548S, V715G)
MDHAEENEIPAETQRYYVERPIFSHPVLQERLHVKDKVTESIGDKLKQAFTCTPK
KIRNIIYMFLPITKWLPAYKFKEYVLGDLVSGISTGVLQLPQGLAFAMLAAVPPVF
GLYSSFYPVIMYCFFGTSRHISIGPFAVISLMIGGVAVRLVPDDIVIPGGVNATNGTE
ARDALRVKVAMSVTLLSGIIQFCLGVCRFGFVAIYLTEPLVRGFTTAAAVHVFTSM
LKYLFGVKTKRYSGIFSVVYSTVAVLQNVKNLNVCSLGVGLMVFGLLLGGKEFN
ERFKEKLPAPIPLEFFAVVMGTGISAGFNLHESYSVDVVGTLPLGLLPPANPDTSLF

HLVYVDAIAIAIVGFSVTISMAKTLANKHGYQVDGNQELIALGICNSIGSLFQTFSI
SCSLSRSLVQEGTGGKTQLAGCLASLMILLVILATGFLFESLPQAVLSAIVIVNLKG
MFMQFSDLPFFWRTSKIELTIWLTTFVSSLFLGLDYGLITAVIIALLTVIYRTQSPSY
KVLGQLPDTDVYIDIDAYEEVKEIPGIKIFQINAPIYYASSDLYSSALKRKTGVNPA
LIMGARRKAMRKYAKEVGNANVANATVVKVDAEVDGENATKPEEEDDEVKFPP
IVIKTTFPEELQRFLPQGENVHTVILDFTQVNFVDSVGVKTLAGIVKEYGDVGIYV
YLAGCSPQVVNDLTRNNFFENPALKELLFHSIHDAVLGSQGREAMAEQEATASLP
QEDMEPNATPTTPEA

MM-Prestin(N548S)

MDHAEENEIPAETQRYYVERPIFSHPVLQERLHVKDKVTESIGDKLKQAFTCTPK
KIRNIIYMFLPITKWLPAYKFKEYVLGDLVSGISTGVLQLPQGLAFAMLAAVPPVF
GLYSSFYPVIMYCFFGTSRHISIGPFAVISLMIGGVAVRLVPDDIVIPGGVNATNGTE
ARDALRVKVAMSVTLLSGIIQFCLGVCRFGFVAIYLTEPLVRGFTTAAAVHVFTSM
LKYLFGVKTKRYSGIFSVVYSTVAVLQNVKNLNVCSLGVGLMVFGLLLGGKEFN
ERFKEKLPAPIPLEFFAVVMGTGISAGFNLHESYSVDVVGTLPLGLLPPANPDTSLF
HLVYVDAIAIAIVGFSVTISMAKTLANKHGYQVDGNQELIALGICNSIGSLFQTFSI
SCSLSRSLVQEGTGGKTQLAGCLASLMILLVILATGFLFESLPQAVLSAIVIVNLKG
MFMQFSDLPFFWRTSKIELTIWLTTFVSSLFLGLDYGLITAVIIALLTVIYRTQSPSY
KVLGQLPDTDVYIDIDAYEEVKEIPGIKIFQINAPIYYASSDLYSSALKRKTGVNPA
LIMGARRKAMRKYAKEVGNANVANATVVKVDAEVDGENATKPEEEDDEVKFPP
IVIKTTFPEELQRFLPQGENVHTVILDFTQVNFVDSVGVKTLAGIVKEYGDVGIYV
YLAGCSPQVVNDLTRNNFFENPALKELLFHSIHDAVLGSQVREAMAEQEATASLP
QEDMEPNATPTTPEA

MM-Prestin(V715G)

MDHAEENEIPAETQRYYVERPIFSHPVLQERLHVKDKVTESIGDKLKQAFTCTPK
KIRNIIYMFLPITKWLPAYKFKEYVLGDLVSGISTGVLQLPQGLAFAMLAAVPPVF
GLYSSFYPVIMYCFFGTSRHISIGPFAVISLMIGGVAVRLVPDDIVIPGGVNATNGTE
ARDALRVKVAMSVTLLSGIIQFCLGVCRFGFVAIYLTEPLVRGFTTAAAVHVFTSM
LKYLFGVKTKRYSGIFSVVYSTVAVLQNVKNLNVCSLGVGLMVFGLLLGGKEFN
ERFKEKLPAPIPLEFFAVVMGTGISAGFNLHESYSVDVVGTLPLGLLPPANPDTSLF
HLVYVDAIAIAIVGFSVTISMAKTLANKHGYQVDGNQELIALGICNSIGSLFQTFSI
SCSLSRSLVQEGTGGKTQLAGCLASLMILLVILATGFLFESLPQAVLSAIVIVNLKG
MFMQFSDLPFFWRTSKIELTIWLTTFVSSLFLGLDYGLITAVIIALLTVIYRTQSPSY

KVLGQLPDTDVYIDIDAYEEVKEIPGIKIFQINAPIYYANSDLYSSALKRKTGVNPA
LIMGARRKAMRKYAKEVGNANVANATVVKVDAEVDGENATKPEEEDDEVKFPP
IVIKTTFPEELQRFLPQGENVHTVILDFTQVNFVDSVGVKTLAGIVKEYGDVGIYV
YLAGCSPQVVNDLTRNNFFENPALKELLFHSIHDAVLGSQGREAMAEQEATASLP
QEDMEPNATPTTPEA
